# Changing winter climate and snow conditions induce various transcriptional stress responses in Scots pine seedlings

**DOI:** 10.3389/fpls.2022.1050903

**Published:** 2022-12-09

**Authors:** Jaana Vuosku, Françoise Martz, Ville Hallikainen, Pasi Rautio

**Affiliations:** ^1^ Natural Resources Unit, Natural Resources Institute Finland, Rovaniemi, Finland; ^2^ Ecology and Genetics Research Unit, University of Oulu, Oulu, Finland

**Keywords:** abiotic stress, circadian clock, climate change, conifer, gene expression, *Pinus sylvestris*, snow cover, stress combination

## Abstract

In northern boreal forests the warming winter climate leads to more frequent snowmelt, rain-on-snow events and freeze-thaw cycles. This may be harmful or even lethal for tree seedlings that spend even a half of the year under snow. We conducted a snow cover manipulation experiment in a natural forest to find out how changing snow conditions affect young Scots pine (*Pinus sylvestris* L.) seedlings. The ice encasement (IE), absence of snow (NoSNOW) and snow compaction (COMP) treatments affected ground level temperature, ground frost and subnivean gas concentrations compared to the ambient snow cover (AMB) and led to the increased physical damage and mortality of seedlings. The expression responses of 28 genes related to circadian clock, aerobic and anaerobic energy metabolism, carbohydrate metabolism and stress protection revealed that seedlings were exposed to different stresses in a complex way depending on the thickness and quality of the snow cover. The IE treatment caused hypoxic stress and probably affected roots which resulted in reduced water uptake in the beginning of the growing season. Without protective snowpack in NoSNOW seedlings suffered from cold and drought stresses. The combination of hypoxic and cold stresses in COMP evoked unique transcriptional responses including oxidative stress. Snow cover manipulation induced changes in the expression of several circadian clock related genes suggested that photoreceptors and the circadian clock system play an essential role in the adaptation of Scots pine seedlings to stresses under different snow conditions. Our findings show that warming winter climate alters snow conditions and consequently causes Scots pine seedlings various abiotic stresses, whose effects extend from overwintering to the following growing season.

## Introduction

Northern boreal forest ecosystems have evolved under the constraints imposed by a short growing season and severe winters during which snow may cover the ground for over a half a year (Gauthier et al., 2015). Thus, winter conditions are of key importance to the function of the ecosystems and may also cause cascading effects throughout the growing season (Matsumura et al., 2010). Despite this, the impacts of changing winter climate and snow cover have received much less attention compared to the effects of climate change during the growing season (Bokhorst et al., 2016). During ongoing climate change, northern boreal forests are predicted to be exposed to greater warming than most other terrestrial biomes and, furthermore, temperatures will increase even more in winter than in summer (Price et al., 2013; Vikhamar-Schuler et al., 2016; Kivinen et al., 2017; Rantanen et al., 2022). Wintertime temperatures and precipitation have a strong impact on the properties of the snowpack. More frequent midwinter warming, and rain-on-snow events lead to partial or complete snow melt, compaction of snowpack and, when freezing returns, formation of ice layers above and within the snowpack as well as ground ice encasement (Ye et al., 2008; Bokhorst et al., 2009).

The research focusing only on growing season climate may overestimate the positive effects of warming temperatures on tree and forest growth in seasonally snow-covered forests (Reinmann et al., 2019). Although evergreen conifers are champions in winter survival (Chang et al., 2021), tree seedlings are vulnerable to abiotic stresses (Grossnickle, 2012) and respond more sensitively to environmental changes than adult trees (Lloret et al., 2009). The establishment, winter survival and growth of young conifer seedlings are strongly depended on the snow cover (Martz et al., 2016; Renard et al., 2016; Domisch et al., 2018). Moreover, evergreen tree seedlings and dwarf shrubs have shown higher mortality than deciduous tree seedlings and grasses after midwinter warming events (Bokhorst et al., 2018). Thus, further warmer winters with changing snow conditions may affect seedling growth and forest productivity, but also change tree species composition and even endanger the natural regeneration of forest.

Snow, as a porous medium with large air content, has a high insulation capacity (Pomeroy and Brun, 2001). Therefore, a snow cover protects effectively underlying vegetation and soil against climatic extremes such as very low temperatures and winter desiccation (Sakai and Larcher, 1987). In spring, snowmelt provides a flush of nutrients and water and thus promotes the start of a growing season (Edwards et al., 2007). However, snow can also act as a stress factor for plants. Under snow, plants suffer light deprivation, although a small portion of the incident solar radiation which is not reflected is absorbed by or transmitted through the snowpack (Robson and Aphalo, 2019). A prolonged snow cover weakens plants by impeding metabolic processes and reduces the length of the growth period (Sakai and Larcher, 1987). On the other hand, high winter and spring temperatures will result in early snowmelt and misleadingly ameliorated environmental conditions which promote dehardening and growth onset thus extending the risk period of frost damage (Beck et al., 2004; Hänninen, 2006; Muilu-Mäkelä et al., 2017). Low oxygen soil conditions caused by compacted snow or ice encasement (Martz et al., 2016) may be detrimental to plants by reducing the efficiency of cellular ATP production and, furthermore, leading to the accumulation of toxic metabolites as a result of the anaerobic metabolism (Fukao and Bailey-Serres, 2004).

Plants have evolved various strategies to resist, tolerate, and recover during the periods of different kind of stresses and stress combinations during their lifespan (Niinemets, 2010). Circadian clock plays an essential role in stress tolerance and acclimation by coordinating the timing of diverse physiological events with the environment (Fowler et al., 2005; Legnaioli et al., 2009; Lai et al., 2012) and controlling the expression of a large group of stress-responsive genes (Sanchez et al., 2011; Grundy et al., 2015). Plants suffering from abiotic stresses commonly face an enhanced accumulation of reactive oxygen species (ROS), which primally act as signal transduction molecules that regulate different pathways during stress acclimation but are also toxic byproducts of stress metabolism (Choudhury et al., 2017). The balance between carbon gain through photosynthesis and carbon loss through respiration is affected by temperature (Atkin and Tjoelker, 2003) and water availability (Pinheiro and Chaves, 2011), and further, carbon status regulates plant growth (Sulpice et al., 2009). Moreover, nonstructural carbohydrates (NSCs), i.e. soluble sugars and starch, together with their associated metabolic enzymes are strongly related to various stress responses and ROS accumulation (Kontunen-Soppela et al., 2002; Yano et al., 2005; Nishizawa et al., 2008; Keunen et al., 2013; ElSayed et al., 2014). Storage form of NSCs, which can be mobilized under stress and disturbance, is thought to be critical for survival, especially for long-lived trees (Palacio et al., 2014).

The aim of this study was to gain a better understanding of snow impact on young conifer seedlings and forest regeneration in northern ecosystems under climate change. We conducted a snow cover manipulation experiment in a natural boreal forest on the Arctic circle to find out how changing snow conditions affect Scots pine at the vulnerable seedling phase. The field experiment was carried out throughout two whole winter periods and growing seasons when Scots pine seedlings were exposed to snow cover manipulation treatments representing three different scenarios of snow conditions in future climate. We hypothesized that (I) ice encasement (IE), the absence of snow cover (NoSNOW) and snow compaction (COMP) expose Scots pine seedlings to different abiotic stresses. Further we aimed to find out if, in addition to immediate stress, (II) abnormal snow conditions cause delayed stress during the early growing season by affecting both the environmental factors and the physiological status of the seedlings. (III) Snow conditions may also have an impact on the growth and health of seedlings during the following summer. In order to answer these questions, we monitored the effects of the snow manipulation treatments on several environmental factors, and further, found out how they affected Scots pine seedlings by studying expression of stress responsive genes, NSC metabolism, photosynthetic activity as well as seedlings’ growth and health.

## Material and methods

### Field experiment and snow cover manipulation treatments

The snow cover manipulation experiment was carried out in a boreal coniferous forest (Tavivaara, 66°25 ‘35”N 25°41’42”E) on the Arctic Circle near Rovaniemi city during the winter periods 2013 – 2014 and 2014 – 2015 (from December to April) (Martz et al., 2016). Ten randomized blocks were positioned into the experimental forest stand. Four rectangle plots (1 m x 3 m) were selected within each block and divided into two subplots (A and B) separated by a 1 m buffer zone in between them (**Supplementary Figure 1**). The plots were randomly assigned to the following treatments: Ambient snow cover (AMB, no snow manipulation treatment), ice encasement (IE, formation of ice layers within the snowpack by watering), no snow (NoSNOW, protection from snow fall and drifted snow by covering the treatment plots) and compacted snow (COMP, snow compaction to reduce the insulating capacity). Snow watering (3,8 l/m^2^) and compaction were conducted three times (December 13, December 17 and January 9) during winter 2013-2014 and twice (January 8 and February 27) during the winter 2014 – 2015 (**Supplementary Figure 2**).

### Environmental factors

Air and soil temperatures, snow depth, snow water equivalent (SWE), soil frost depth and the depth of upper unfrozen ground as well as oxygen (O_2_) and carbon dioxide (CO_2_) concentrations in soil were measured in the treatment plots as previously described in Martz et al. (2016).

### Plant material

Scots pine (*Pinus sylvestris* L.) is the most widely distributed Eurasian conifer and one of the keystone species in the Eurasian boreal forest zone, growing in many ecologically diverse habitats (Mirov, 1967; Durrant et al., 2016). Especially in the northern part of its distribution, Scots pine as a dominant tree in many forested areas plays an essential role in forest ecosystems function (Durrant et al., 2016; Klimek et al., 2016). In September 2013, 800 one-year-old Scots pine seedlings (supplied by Fin Forelia Oy, Rovaniemi, Finland), whose height varied between 7 and 12 cm, were planted to the experiment field with 10 seedlings per subplot. In total, the density of conifer seedlings was 20 seedlings/m2 (10 Scots pine and 10 Norway spruce (*Picea abies* (L.) Karst) seedlings) in all plots including the plots without a snow cover manipulation treatment (**Supplementary Figure 1**).

The seedlings had grown from wild-collected local seeds, planted in containers filled with fertilized and limed sphagnum peat. The health and annual growth of the seedlings were recorded after one winter of snow manipulation during the growing season 2014 (Martz et al., 2016) after which seedlings were exposed to the second round of snow manipulation treatments during the winter 2014 – 2015. In spring 2015, the first sampling was conducted from 4 to 7 May (the first sampling date), when soil was still frozen and hence the seedlings were drilled off the ground. The second sampling was carried out between 8 and 11 June (the second sampling date). The samplings were timed between 10am and 3pm. In each block all treatment plots were collected all at once and processed immediately. In each treatment plot, the current needles (i.e. needles formed during the previous growing season) were excised from one healthy looking-seedling, frozen immediately in liquid nitrogen and stored at −80°C for gene expression and NSC analyses. Also, stems (−80°C) and roots (−20°C) were cold-stored for the NSC analyses. For the measurement of chlorophyll fluorescence, current needles were detached from the stem in the field, wrapped in aluminium foil and stored in an icebox containing wet paper and cooling blocks and measured on the same day. In addition, on the first sampling date, the apical bud of each seedling was cut, and tissues were fixed immediately (2% glutaraldehyde in cacodylate buffer, pH 7.0) for the microscopical observation.

### Gene selection

Stress defense and acclimation responses in Scots pine seedlings under the snow cover manipulation treatments were studied by gene expression analyses using real-time quantitative PCR (qPCR). The selection of the genes was based on their stress related functions and/or previously observed stress responses in the transcriptome profiling studies in conifers. Altogether, 28 genes related to carbohydrate metabolism (*AMY1, βG, B3GALT, BMY1, GOLS2, SEX1, SPS, SS3, SUS*, *WAXY1*), anaerobic metabolism (*ADH, PDC, PFP, PK*), photosynthesis (*RBCS*), photorespiration (*MSHMT*), dehydration and cold stresses (*DHN3*, *LEA*), ROS scavenging (*CAT*, *SOD*), polyamine biosynthesis (*ADC*), autophagy (*ATG8*) and circadian clock (*CRY1*, *CRY2*, *GI*, *PHYN*, *PHY*O, *TOC*1) were studied (**Table 1**).

**Table 1:** Genes used for studying stress defense and acclimation responses in Scots pine seedlings under snow cover manipulation treatments.

Gene	Protein	Function	References
*ADC*	Arginine decarboxylase (EC 4.1.1.19)	Putrescine biosynthesis	Tiburcio et al., 2014; Muilu-Mäkelä et al., 2015
*ADH*	Alcohol dehydrogenase (EC 1.1.1.1)	Anaerobic fermentation	Jarillo et al., 1993; Watkinson et al., 2003
*AMY1*	Alpha-amylase 1 (EC 3.2.1.1)	Starch hydrolysis	Holliday et al., 2008; Thalmann and Santelia, 2017
*ATG8*	Autophagy-related protein 8	Autophagy	Sláviková et al., 2005; Salo et al., 2016
*βG*	Beta-glucosidase (EC 3.2.1.21)	Hydrolysis of monolignol glucosides	Lee et al., 2007; Holliday et al., 2008; Khazaei et al., 2015; Vuosku et al., 2015
*B3GALT*	Beta-1,3-galactosyltransferase	Protein N-glycosylation	Holliday et al., 2008; Nagashima et al., 2018
*BMY1*	Beta-amylase 1 (EC 3.2.1.2)	Starch hydrolysis	Behringer et al., 2015; Zanella et al., 2016; Thalmann and Santelia, 2017
*CAT*	Catalase (EC 1.11.1.6)	Conversion of H_2_O_2_ into water and molecular oxygen	Holliday et al., 2008; Mhamdi et al., 2010; Vuosku et al., 2015
*CRY1*	Cryptochrome 1	Blue light receptor	Ma et al., 2016; Alakärppä et al., 2018
*CRY2*	Cryptochrome 2	Blue light receptor	Alakärppä et al., 2018; D’Amico-Damião and Carvalho, 2018
*DHN3*	Dehydrin 3	Dehydration, cold and freezing stress	Holliday et al., 2008; Hanin et al., 2011
*GI*	Gigantean	Circadian clock, carbohydrate metabolism, cold stress response	Holliday et al., 2008; Mishra and Panigrahi, 2015; Alakärppä et al., 2018
*GOLS2*	Galactinol synthase 2	Raffinose biosynthesis	Nishizawa et al., 2008; Holliday et al., 2008; Behringer et al., 2015
*LEA*	Late embryogenesis abundant protein	Desiccation tolerance	Watkinson et al., 2003; Holliday et al., 2008; Behringer et al., 2015
*MSHMT*	Mitochondrial serine hydroxymethyltransferase (EC 2.1.2.1)	Photorespiration	Moreno et al., 2005; Voss et al., 2013
*PDC*	Pyruvate decarboxylase (EC 4.1.1.1)	Ethanolic fermentation	Kürsteiner et al., 2003; Watkinson et al., 2003
*PFP*	Pyrophosphate–fructose-6-phosphate 1-phosphotransferase (EC 2.7.1.90)	Glycolysis, Gluconeogenesis	Lim et al., 2014
*PHYN*	Phytochrome N	Red and far-red light photoreceptor	Holliday et al., 2008; Carvalho et al., 2011; Alakärppä et al., 2018
*PHYO*	Phytochrome O	Red and far-red light photoreceptors	Carvalho et al., 2011; Alakärppä et al., 2018
*PK*	Pyruvate kinase (EC 2.7.1.40)	Glycolysis	Watkinson et al., 2003
*RBCS*	Ribulose-1,5-bisphosphate carboxylase/oxygenase small subunit (RUBISCO; EC 4.1.1.39)	Photosynthesis	Dubos et al., 2003; Watkinson et al., 2003
*SEX1*	Alpha-glucan water dikinase 1, also called starch-related R1 protein (GWD; EC 2.7.9.4)	Phosphorylation of glucosyl residues of starch	Yano et al., 2005; Mahlow et al., 2016; Thalmann and Santelia, 2017
*SOD*	CuZn superoxide dismutase (EC 1.15.1.1)	ROS scavenging	Anderson et al., 1992; del Río et al., 2018
*SPS*	Sucrose-phosphate synthase (EC 2.4.1.14)	Sucrose biosynthesis	Dubos et al., 2003
*SS3*	Starch synthase 3 (EC 2.4.1.21)	Starch biosynthesis	Thalmann and Santelia, 2017
*SUS*	Sucrose synthase (SUS; EC 2.4.1.13)	Sucrose metabolism	Watkinson et al., 2003; Dubos et al., 2003; Holliday et al., 2008
*TOC1*	Timing of CAB expression 1	Core oscillator component of circadian clock	Legnaioli et al., 2009; Lai et al., 2012; Alakärppä et al., 2018
*WAXY1*	Granule-bound starch synthase 1 (EC 2.4.1.242)	Starch biosynthesis	Thalmann and Santelia, 2017

### RNA extraction and reverse transcription

Gene expression was studied in needles, which in conifers are the key perennial organ that integrates daily and seasonal signals from light, temperature, and water availability (Cronn et al., 2017). Total RNA was extracted from ten biological replicates for each treatment and sampling date using the KingFisher™ ml method (Thermo Electron Corporation) with KingFisher™ Pure RNA Plant Kit (Thermo Fisher Scientific) according to the manufacturer’s instructions. The extraction procedure included a DNase treatment. Needles were homogenized in liquid nitrogen using a mortar and pestle before the RNA extraction. The RNA yields were measured twice with the Qubit Fluorometer (Invitrogen) using a Qubit^®^ RNA HS Assay Kit (Life Technologies) and RNA integrity was checked by agarose gel electrophoresis. cDNA synthesis was performed from 200 ng of total RNA in a reaction volume of 20 *μ*L using qScript cDNA Supermix containing random hexamers and oligo-dT primers (Quanta Biosciences).

### Quantitative PCR

The qPCR amplification conditions were optimized for the CFX96™ Real-time PCR detection system (Bio-Rad) to achieve the PCR efficiency of at least 1.8. The subsequent PCR runs showed a single PCR product with the expected size during the melting curve and electrophoretic analyses. PCR amplification was performed using the SsoFast™ EvaGreen^®^ Supermix kit (Bio-Rad), 50 nM gene-specific primers (**Supplementary Table 1**) and 2 μl cDNA (1:10 dilution) in the reaction volume of 20 μl. PCR amplification was initiated by incubation at 95°C for 3 min followed by 40 cycles: 10 s at 95°C, 10 s at 58°C, and 20 s at 72°C. A melting curve analysis to confirm specific amplification was conducted by stepwise temperature increase from 65°C to 95°C at 0.5°C/step. The arithmetic mean of two technical replicates was used in the data analysis. The geometric mean of expression level in three independently regulated reference genes (Vandesompele et al., 2002) *actin* (*ACT*), *ubiquitin* (*UBI*) and *glyceraldehyde-3-phosphate dehydrogenase* (*GAPDH*) from different functional classes was used for the normalization of the gene expression levels according to the Pfaffl (2001) method.

### Non-structural carbohydrates

Frozen needles, stems and roots were freeze-dried (FreeZone 4.5 L -84°C, Labconco) and ground into fine powder with a bead homogenizer (Precellys24, Bertin Instruments). Subsequently, 15 mg (needles) or 20 mg (stems and roots) of dry powder was used for NSC analyses as previously described in detail by Domisch et al. (2018). Glucose, fructose, sucrose, pinitol, raffinose and xylose were quantified using known standards by high-performance liquid chromatography (HPLC; Shimadzu, NexeraX2) using a ligand-exchange column (Hi-Plex Ca, 300 x 7.7 mm, Agilent) and a guard column (Hi-Plex Ca 50 x 7.7 mm, Agilent). Starch was enzymatically hydrolyzed and glucose was quantified using the glucose oxidase/peroxidase (GOPOD) kit (K-GLUC, Megazyme) in a microplate format (MultiSkan FC, Thermo Scientific).

### Chlorophyll fluorescence

About 10 needles were collected from each seedling in the field, wrapped into an aluminum foil and stored in a moist ice box until measurement after a maximum of 6 hours. After equilibration to room temperature, needles were placed on a black adhesive tape the upper side facing up. The maximum and effective photosynthetic quantum efficiency was measured from dark acclimated (20 min) needles using a portable fluorometer (Walz PAM-2500, Heinz Walz GmbH, Effeltrich, Germany). A control experiment showed that storage in moist ice box (+4°C) for up to 20h had no effect on the chlorophyll fluorescence measured from dark- or light acclimated needles (data not shown).

### Seedlings’ growth and health

Scots pine seedlings form apical buds, also known as terminal buds, in late summer in response to the shortening photoperiod and decreasing temperature which terminate growth. After a period of cold temperatures in winter, increasing light and temperature in spring induce bud swelling which precedes bud burst (opening of bud scales) and growth of new needles (Cooke et al., 2012; Avia et al., 2014). The apical buds collected on the first sampling date were examined under a stereomicroscope (Wild Heerbrugg) and photographed using an Infinity 1 microscopy camera (Lumenera Corporation) to evaluate damage caused by the snow manipulation treatments during winter. The buds were classified as healthy, slightly damaged or heavily damaged (**Supplementary Figure 3**). During the growing season 2015, the growth and health of the main shoot in the remaining eight seedlings (see **Supplementary Figure 1**) were recorded three times: in the beginning (June 8), in the middle (July 15) and after the cessation of the annual growth in the end of the growing season (October 1). The length of the 2015 annual growth of the main shoot was measured. In few cases when the apical bud was dead and the secondary buds represented a source of new growth the length of the shoots originating from these secondary buds was measured. The shoots were categorized into four classes according to the proportion of brown needles: 1. no brown needles i.e. healthy seedling, 2. proportion of brown needles < 50%, 3. proportion of brown needles > 50% and 4. dead shoot. The growth was measured only in living seedlings having some green needles left.

### Statistical analyses

Linear mixed effects models were constructed for analyzing the gene expression, chlorophyll fluorescence, NSC, growth and health data. In the randomized block design, the hierarchy in the data was as follows: a seedling nested within a square 1×1m (sub)plot (representing one of the four treatments), and the square plots nested within the blocks. Thus, there were four square 1×1m (sub) plots in each of the 10 blocks. One seedling was randomly sampled out from each square plot in May and another seedling in June for the gene expression analyses. The relative expression of each gene was analyzed separately using a linear mixed effects model. The tested model consisted of sampling date, treatment and their interaction effect as fixed factors. The random part of the model consisted of seedlings nested within square (sub)plots nested within the blocks. The correlation structure was assumed as compound symmetry. The response variable in the models was the relative expression of a gene. The residuals were considered as normally or log-normally distributed. The models were tested with and without the log-transformation, and the better version was selected as the final model. The chlorophyll fluorescence (response Fv/Fm) model had the similar structure than the gene models considering the fixed and random part of the model.

Separate linear mixed effects models were also constructed for the NSC concentrations. The tested fixed variables in the models were: sampling date, treatment, organ (categories needles, stems and roots) and all their two-way and three-way interaction effects. Thus, we tested the hypothesis based on the full model, similarly to the gene models. The random parts of the NSC models were like in the gene models, but there were three measurements (carbohydrate concentrations in needles, stem and roots) from each of the seedlings. Thus, we tested two different correlation structures of the residual variance, compound symmetry and autoregressive (AR-1) structure, because a spatial correlation could be expected between the needles, stem and roots. In the final model, the significantly (at 5% risk level) better (based on the difference in log-likelihood value of the models) correlation structure was used. Furthermore, the log-transformation for the response variables (carbohydrate concentrations) was used if it produced better residuals of the models. In addition to the general linear NSC models, a model was constructed for the proportion of non-soluble (starch) sugar and the total sugar content (sum of soluble and non-soluble sugars). The model was constructed similarly as the NSC models described above, but the response variable (the proportion) was considered as binomially distributed, and the generalized linear mixed model using binomial distribution assumption and logit link function was used instead of the traditional arcsin- square root transformations to the response variable (e.g. Crawley, 2007; Miina et al., 2009). The dispersion parameter was estimated in this logistic model (not fixed as 1).

In the model constructed for the height increment, the hierarchical structure of the random part was like the model described earlier, but each of the seedlings was measured three times during the growing season. Thus, the design included autoregressive (AR-1) repeated measurements in addition to the hierarchical design. The height increments were log-transformed for the modeling. In the model, the fixed variables that were tested were date of the measurement (June, July, October), treatment and their interaction effects. The log-transformed responses were back transformed by adding the halfs of the variances (different hierarchical levels) to the predicted values in the log-transformed scale and making the exponential transformation to these values (e.g. Lappi, 1993).

The linear mixed effects models were also computed to test the effects of four environmental variables (mean snow depth on March 19, mean snow water equivalent on April 13 (SWE) and the mean concentrations of O_2_ and CO_2_ in April) on the expression of three selected genes (*DHN3*, *GOLS2* and *PCD*) on the firs sampling date. The environmental variables were tested separately, because of their strong positive or negative correlation with each other. The distributions of the gene expression values were log-transformed for the analysis to get more normally distributed error term. The mixed effects linear model allowed the use of the block as a random factor in the models. Compound symmetry was assumed as the correlation structure for the random blocks (G-matrix).

The canonical correspondence analysis (CCA) was performed for studying the association between the gene expression data on the first sampling date and the snow manipulation treatments using eight environmental variables: mean ground temperature in January and April, mean snow depth on March 19, mean snow water equivalent on April 13, mean soil frost depth in February, the mean depth of unfrozen soil surface from April 22 to May 20 and the mean concentrations of O_2_ and CO_2_ in April.

The gene expression heatmaps with and without the hierarchical clustering option were created with the heatmap.2 function in the gplots package (Warnes et al., 2016) using R version 3.4.2 (R Core Team, 2013). The general linear mixed models were computed using R package nlme (Pinheiro et al., 2020) and R package MASS (Venables and Ripley, 2002) with function glmmPQL, and SPSS ver. 26. The prediction plots were constructed using R package effects (Fox, 2003).

## Results

### Snow conditions altered environmental factors

The snow manipulation treatments had clear impacts on the observed environmental factors during the winters 2013-2014 and 2014-2015. The ground surface temperature was close to the air temperature in NoSNOW, and the temperature decrease was noticeably also in IE and COMP compared to AMB (Martz et al., 2016 and **Supplementary Figure 4**). In 2014-2015, snow depth was lower, and snow water equivalent higher in IE and COMP compared to AMB (**Supplementary Figure 5**). In the end of February, when soil frost was at deepest, the IE and COMP treatments caused about 10 cm deeper soil frost than AMB, whereas in NoSNOW the lack of the insulating snow cover resulted in about half a meter deeper soil frost compared to AMB (**Supplementary Figure 6A**). On the other hand, close to the soil surface frost started to thaw earlier in the NoSNOW, IE and COMP treatments than in AMB (**Supplementary Figure 6B**). Snow manipulation had also an effect on soil gases as O_2_ decreased and CO_2_ increased under the IE and COMP treatments so that O_2_ concentrations were below 15% over a month during the spring (**Supplementary Figure 7**). In 2013-2014 the effect of snow manipulation to soil gases was even stronger (Martz et al., 2016).

### Snow conditions caused both immediate and subsequent gene expression responses

**Figure 1:**
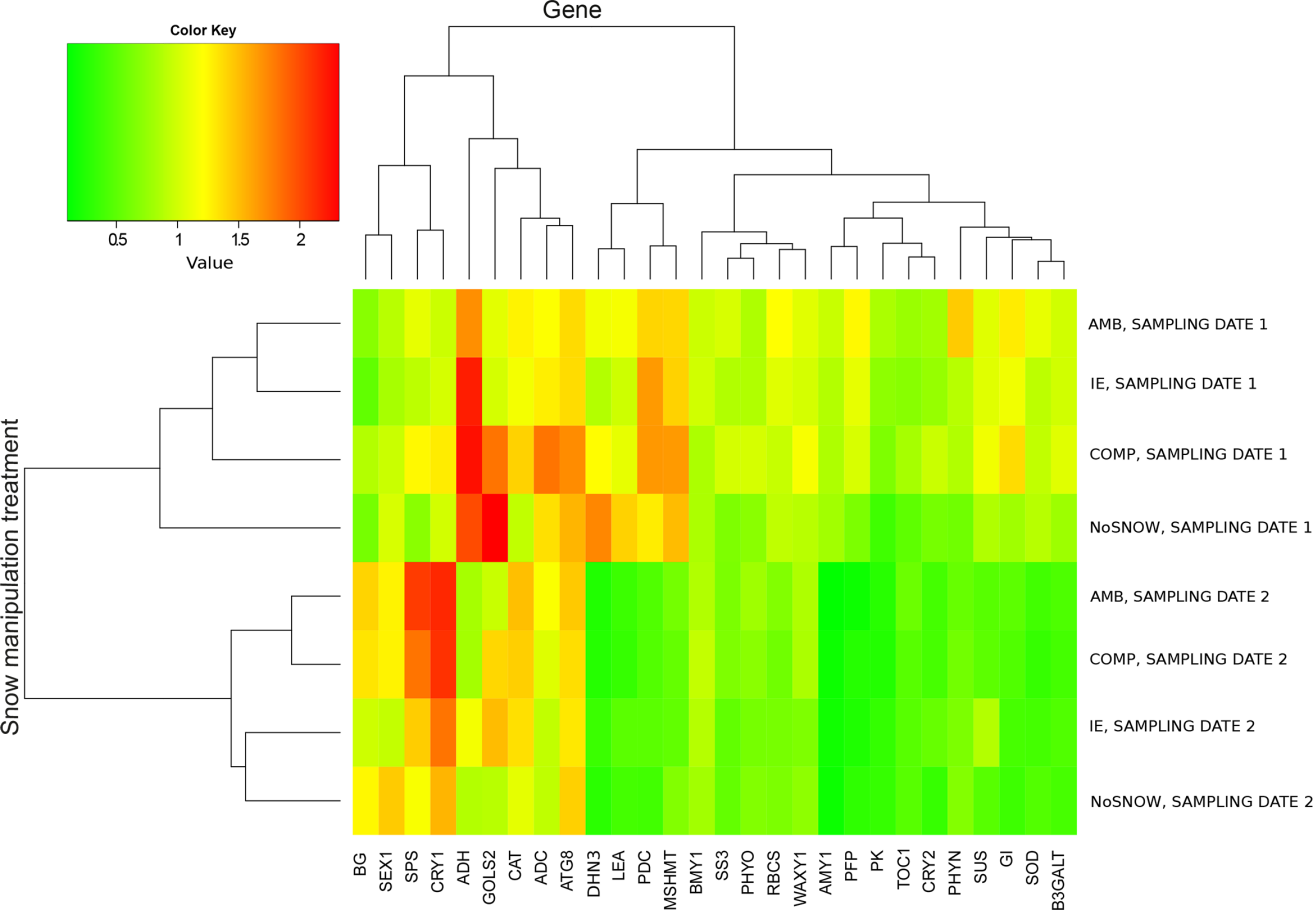
Cluster heatmap of relative gene expression in Scots pine seedlings. The genes and snow cover manipulation treatments were grouped on the grounds of mean relative gene expression on the first and second sampling dates using hierarchical clustering (n=10). AMB, ambient snow cover; IE, ice encasement; NoSNOW, no snow; COMP, compacted snow.

In order to find out what kind of stresses Scots pine seedlings experienced in the snow manipulation treatments we studied the expression of 28 genes in needles (**Table 1**). The snow manipulation treatments divided into two groups according to the sampling date in the cluster heatmap of relative gene expression, which indicated extensive changes in gene expression between the sampling dates (**Figure 1**). On the first sampling date, when there was still snow and soil frost, gene expression differed the least between IE and AMB, and between NoSNOW and AMB it differed the most. The expression profiles were characterized by strong expression of cold, drought anaerobic and oxidative stress related genes (*ADC*, *ADH*, *ATG8*, *CAT*, *DHN3*, *GOLS2*, *LEA*, *MSHMT* and *PDC*) variously in all the seedlings but especially under the NoSNOW and COMP treatments. On the second sampling date, the expression of the stress related genes was generally downregulated. Gene expression under the AMB and COMP treatments resembled each other, whereas the IE and NoSNOW treatments clustered together (**Figure 1**).

Other patterns appeared in the heatmap when the mean expression of each gene in IE, NoSNOW and COMP was presented with relation to its expression in AMB (**Supplementary Figure 8**). The target genes were manually grouped on the grounds of their function (carbohydrate metabolism, energy metabolism, stress protection and circadian clock). On the first sampling date, the expression profile in NoSNOW was characterized by downregulation of carbohydrate metabolism related genes, except for *GOLS2*, as well as glycolysis and the circadian clock related genes. On the second sampling date, the expression of the circadian clock genes was generally decreased in IE and NoSNOW compared to AMB. Furthermore, the expression profile of IE was characterized by the responses of several drought and osmotic stress related genes such as *SUS*, *GOLS2, WAXY1*, *SS3, AMY1, DHN3* and *LEA* (**Supplementary Figure 8**).

### Snow conditions affected carbohydrate and energy metabolism, stress protection and circadian clock related gene expression

**Figure 2:**
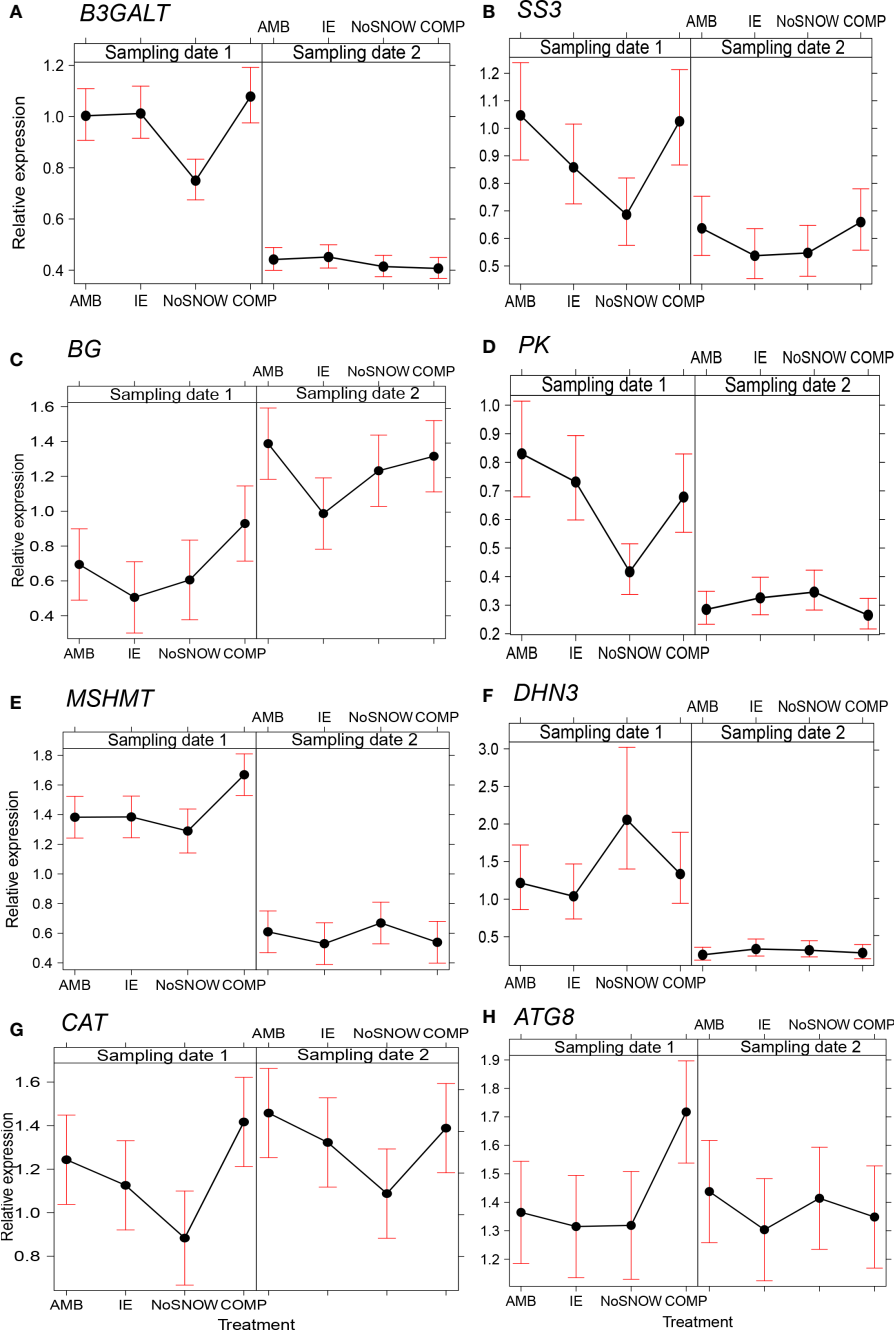
Effects of snow manipulation treatments on *B3GALT*, *SS3*, *βG*, *PK*, *MSHMT*, *DHN3*, *CAT* and *ATG8* expression in Scots pine seedling. The relative expression of the **(A)** beta-1,3-galactosyltransferase (*B3GALT*), **(B)** starch synthase 3 (*SS3*), **(C)** beta-glucosidase (*βG*), **(D)** pyruvate kinase (*PK*), **(E)** mitochondrial serine hydroxymethyltransferase (*MSHMT*), **(F)** dehydrin 3 (*DHN3*), **(G)** catalase (*CAT*) and **(H)** autophagy-related protein 8 (*ATG8*) genes in needles on the sampling dates 1 and 2. AMB, ambient snow cover; IE, ice encasement; NoSNOW, no snow; COMP, compacted snow.

The expression of 14 genes was significantly altered between the snow manipulation treatments (**Table 2**). Among them were three carbohydrate metabolism related genes (**Figures 2A–C**). In NoSNOW the expression of *B3GALT* was downregulated relative to all other treatments (**Figure 2A**) and the expression of *SS3* was downregulated relative to AMB and COMP (**Figure 2B**) on the first sampling date. Additionally, the expression of *βG* was downregulated in IE relative to AMB on the second sampling date (**Figure 2C**). The expression of the following energy metabolism and/or stress protection related genes was altered on the first sampling date (**Figures 2D–H**). Glycolysis related *PK* was downregulated in NoSNOW relative to the other treatments (**Figure 2D**). Photorespiration related *MSHMT* was upregulated in COMP relative to the other treatments (**Figure 2E**), whereas drought and cold stress related *DHN3* was upregulated in NoSNOW (**Figure 2F**). The expression of H_2_O_2_ scavenger *CAT* differed between NoSNOW and COMP in which *CAT* was down- and up-regulated, respectively (**Figure 2G**). Autophagy related *ATG8* were upregulated in COMP relative to the other treatments (**Figure 2H**).

**Table 2:** Effects of sampling date and snow manipulation treatments on gene expression in Scots pine seedlings.

Gene	Sampling date	Snow manipulation treatment	Interaction	Gene	Sampling date	Snow manipulation treatment	Interaction
*SUS* (log)	20.975 (1) p < 0.001	1.569 (3) p = 0.666	3.136 (3) p = 0.371	*RBCS* (log)	10.152 (1) p = 0.001	0.662 (3) p = 0.882	0.250 (3) p = 0.969
*SPS*	15.625 (1) p < 0.001	4.439 (3) p = 0.218	3.153 (3) p = 0.369	***MSHMT***	60.076 (1) p < 0.001	15.918 (3) p = **0.001**	13.512 (3) p = **0.004**
*GOLS2* (log)	0.062 (1) p = 0.803	6.151 (3) p = 0.105	5.458 (3) p = 0.141	*LEA* (log)	46.840 (1) p < 0.001	1.460 (3) p = 0.692	3.929 (3) p = 0.269
***B3GALT*** (log)	132.726 (1) p < 0.001	28.827 (3) p < **0.001**	14.068 (3) p = **0.003**	***DHN3*** (log)	54.223 (1) p < 0.001	9.240 (3) p = **0.026**	5.587 (3) p = 0.134
*WAXY1*	3.519 (1) p = 0.061	6.901 (3) p = 0.075	1.494 (3) p = 0.684	***CAT***	2.418 (1) p = 0.314	13.433 (3) p = **0.004**	1.993 (3), p = 0.574
***SS3*** (log)	17.400 (1) p < 0.001	15.207 (3) p = **0.002**	3.089 (3) p = 0.378	*SOD* (log)	51.786 (1) p < 0.001	3.558 (3) p = 0.313	7.522 (3) p = 0.057
*AMY1* (log)	91.881 (1) p < 0.001	0.759 (3) p = 0.859	3.353 (3) p = 0.340	*ADC* (log)	0.018 (1) p = 0.893	4.623 (3) p = 0.202	2.651 (3) p = 0.449
*BMY1* (log)	0.804 (1) p = 0.370	0.662 (3) p = 0.882	0.457 (3) p = 0.928	***ATG8***	0.331 (1) p = 0.565	13.720 (3) p = **0.003**	8.534 (3) p = **0.036**
*SEX1*	7.068 (1) p = 0.008	1.850 (3) p = 0.604	4.737 (3) p = 0.192	***G1*** (log)	44.889 (1) p < 0.001	20.658 (3) p < **0.001**	4.737 (3) p = 0.192
***BG***	22.855 (1) p < 0.001	8.651 (3) p = **0.034**	2.660 (3) p = 0.447	***CRY1*** (log)	55.952 (1) p < 0.001	9.966 (3) p = **0.019**	4.738 (3) p = 0.192
***PK*** (log)	64.466 (1) p < 0.001	27.803 (3) p < **0.001**	24.845 (3) p < **0.001**	***CRY2*** (log)	24.831 (1) p < 0.001	9.309 (3) p = **0.025**	3.956 (3) p = 0.266
*PFP* (log)	28.484 (1) p < 0.001	4.664 (3) p = 0.198	6.049 (3) p = 0.109	***TOC1*** (log)	2.172 (1) p = 0.141	12.025 (3) p = **0.007**	6.007 (3) p = 0.111
*PDC* (log)	82.223 (1) p < 0.001	6.517 (3) p = 0.089	1.475 (3) p = 0.688	***PHYO***	0.651 (1) p = 0.420	22.904 (3) p < **0.001**	12.356 (3) p = **0.006**
*ADH* (log)	29.779 (1) p < 0.001	2.599 (3) p = 0.458	1.348 (3) p = 0.718	***PHYN*** (log)	19.875 (1) p < 0.001	17.367 (3) p = **0.001**	12.127 (3) p = **0.007**

Analysis of deviances for the hypothesis tested (sampling date and snow manipulation treatment as the main effects and their interaction) for gene expression. The chi-squared values, the degrees of freedom (in the parenthesis) and the corresponding p-values are presented. If the response in the model is log-transformed, log is mentioned in the parenthesis. The genes with significant (< 5% risk level) treatment effect and/or its interaction with sampling date are printed in bold.

**Figure 3:**
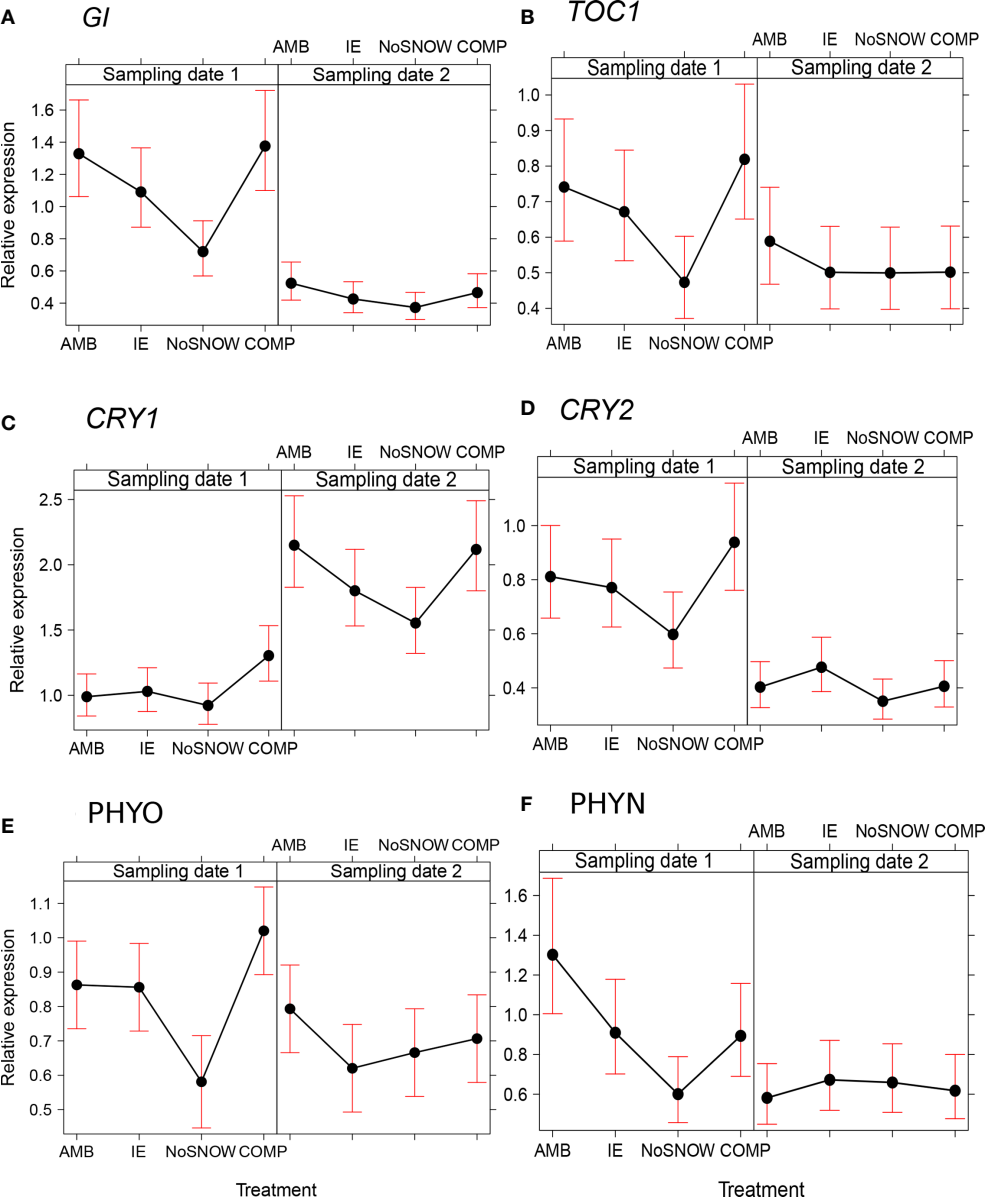
Effects of snow manipulation treatments on the expression of circadian clock genes in Scots pine seedling. The relative expression of the **(A)** gigantean (*GI*), **(B)** timing of CAB expression 1 (*TOC*), **(C)** cryptochrome 1 (*CRY1*), **(D)** cryptochrome 2 (*CRY2*), **(E)** phytochrome O (*PHYO*) and **(F)** phytochrome N (*PHYN*) genes in needles on the sampling dates 1 and 2. AMB, ambient snow cover; IE, ice encasement; NoSNOW, no snow; COMP, compacted snow.

The expression of the circadian clock genes was significantly altered between the snow manipulation treatments on the first sampling date, but the differences leveled off, except for *CRY1*, by the second sampling date (**Figures 3A–F**). The expression of the core clock components, *GI* and *TOC1*, varied consistently between the treatments, when both genes were downregulated in NoSNOW relative to AMB and COMP (**Figures 3A, B**). Also, the expression the photoreceptor genes differed under the treatments. On the first sampling date, *CRY1* was upregulated in COMP, whereas on the second sampling date, *CRY1*was downregulated in NoSNOW relative to AMB and COMP (**Figure 3C**). *CRY2* was slightly downregulated in NoSNOW and slightly upregulated in COMP, which resulted in significant difference between those treatments (**Figure 3D**). *PHYO* was downregulated in NoSNOW relative to the other treatments (**Figure 3E**), whereas *PHYN* was downregulated in NoSNOW relative to AMB (**Figure 3F**).

### Gene expression was associated with environmental factors

We studied the association between gene expression and the environmental factors on the first sampling date. The effects of snow depth, SWE and soil O_2_ and CO_2_ concentrations on the expression of the *DHN3*, *GOLS2* and *PDC* genes on the first sampling date were studied by linear mixed effects models and CCA (**Table 3**; **Supplementary Figure 9**). These genes were selected because the expression of *DHN3* and *GOLS2* generally increases in plants as a response to cold and drought stresses (Holliday et al., 2008; Hanin et al., 2011), whereas *PDC* expression increases specifically under anaerobic stress (Kürsteiner et al., 2003; Watkinson et al., 2003). We found that *GOLS2* expression decreased along with the increasing snow depth, SWE and CO_2_ concentration, whereas it increased along with the increasing O_2_ concentration. Also, *DHN3* expression decreased along with the increasing snow depth and SWE. The CCA ordination plot showed that strong *PDC* expression was associated with high values of snow depth, SWE and CO_2_ concentration, but in the linear mixed effects model a significant coefficient was observed only for SWE (**Table 3**; **Supplementary Figure 9**).

**Table 3:** The effects of snow depth, snow water equivalent (SWE), soil oxygen (O_2_) and carbon dioxide (CO_2_) concentrations on the expression of the dehydrin 3 (*DHN*3), galactinol synthase 2 (*GOLS2*) and pyruvate decarboxylase (*PDC*) genes.

Environmental factor	*DHN3*	*GOLS2*	*PDC*
Snow depth	-**0.015** **p = 0.001**	**-0.022** **p = 0.010**	0.007 p = 0.093
SWE	-**0.001** **p = 0.004**	**-0.002** **p = 0.032**	**0.001** **p = 0.035**
O_2_	0.028 p = 0.115	**0.078** **p = 0.011**	-0.019 p = 0.214
CO_2_	-0.039 p = 0.325	**-0.178** **p = 0.009**	0.038 p = 0.258

The environmental variables correlate strongly with each other, and they were tested separately in the linear mixed effects models (block as a random effect, df=28). Log transformations were made for *DHN3* and *GOLS2* to normalize the distributions. The coefficients of the models with their p-values (significant printed in bold) are presented.

**Figure 4:**
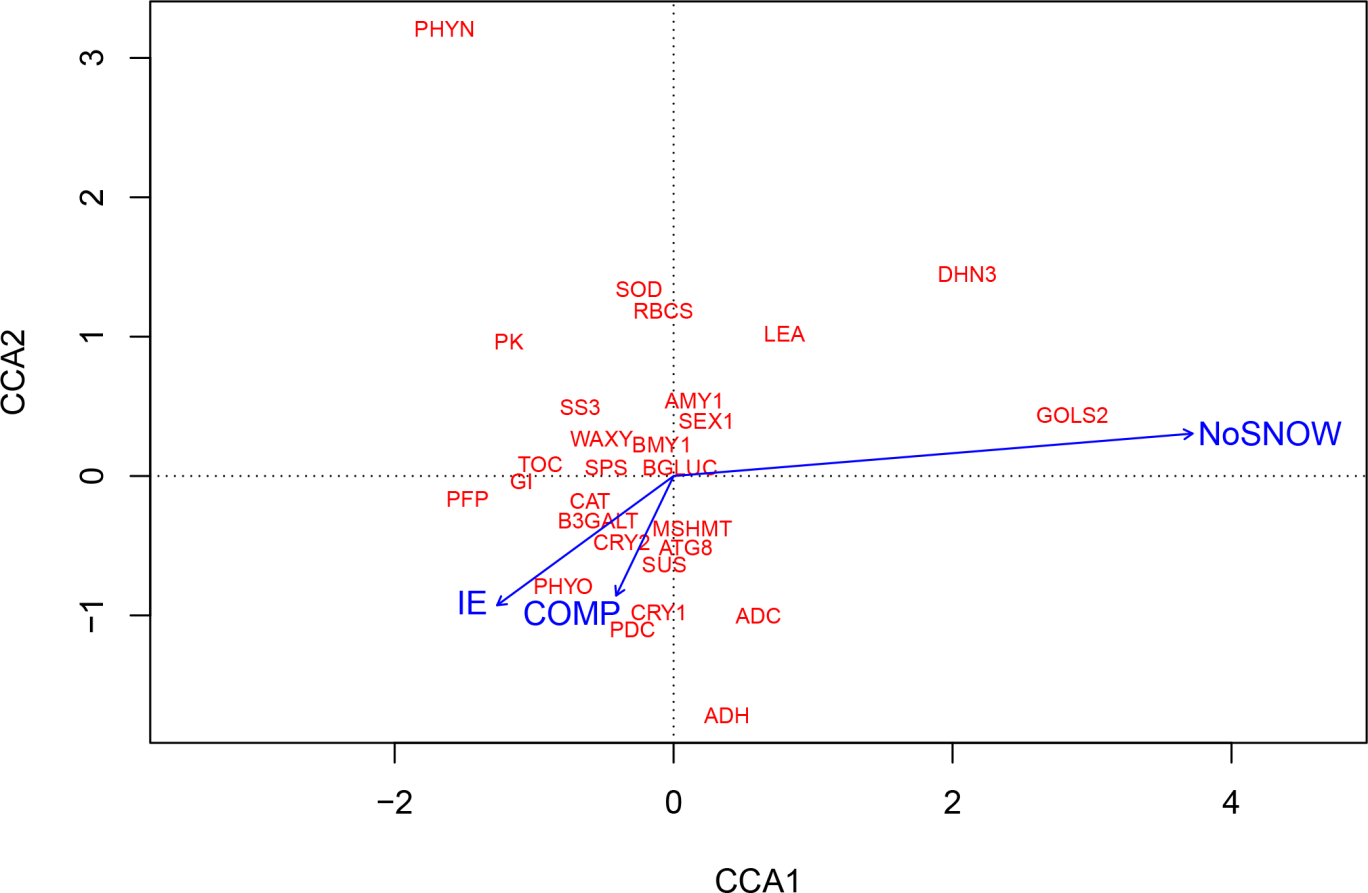
Canonical correspondence analysis (CCA) showing the association between gene expression on the first sampling date and snow manipulation treatments. IE, ice encasement; NoSNOW, no snow; COMP, compacted snow.

CCA was also used for exploring the relationships between gene expression and the snow manipulation treatments, which each represented a bundle of the eight observed environmental variables. The CCA ordination plot shows that the IE, NoSNOW and COMP treatments resulted in very different environmental conditions and, further, induced different gene expression responses in Scots pine seedlings (**Figure 4**). The longest arrow representing the NoSNOW treatment differed most, whereas the IE and COMP arrows were near each other’s. The NoSNOW treatment was associated with the high values in the expression of the *GOLS2*, *DHN3* and *LEA* genes, whereas e.g. *PK*, *PFP*, *GI*, *TOC* and *SS3* were placed in the opposite direction. High *B3GALT, CAT, PDC, MSHMT, ATG8, CRY1, CRY2* and *PHYO* expression was most likely found under the COMP treatment (**Figure 4**).

### NSC concentrations remained consistent under different snow conditions

In Scots pine seedlings NSC concentrations were affected by both the organ and the sampling date, whereas the snow manipulation treatments had a minor effect. Only interactions organ x treatment and organ x sampling date x treatment were significant for starch and pinitol (**Table 4**). On the first sampling date, the NSC pools of needles, stems and roots consisted mostly of soluble sugars and the proportion of starch was small. The proportion of starch was the greatest in roots, 23% of the NSC pool (**Supplementary Figure 10A**). On the second sampling date, the amount of starch increased in all the tissues and proportion of starch was the highest in needles and roots, in which it encompassed 60% and 68% of the NSC pool, respectively (**Supplementary Figures 10A, B**). On the first sampling date, the NSC profiles were characterized by the accumulation of glucose, fructose and pinitol in needles and raffinose in roots (**Supplementary Figures 10C–F**). The sucrose concentrations were higher in stems than in needles and roots on both sampling dates (**Supplementary Figure 10G**). The xylose concentrations were found to be the lowest in stems on the first sampling date, whereas stems had the highest xylose concentrations on the second sampling date (**Supplementary Figure 10H**).

**Table 4:** Effects of organ, sampling date and snow manipulation treatments on nonstructural carbohydrate (NSC) concentrations in Scots pine seedlings.

NSC	Scots pine organ	Sampling date	Treatment	Organ × sampling date	Organ × treatment	Sampling date × treatment	Organ × sampling date × treatment
**Starch** (log)	47.510 (2) p < 0.001	54.079 (1) p < 0.001	0.233 (3) p = 0.972	23.800 (2) p < 0.001	31.970 (6) **p < 0.001**	1.097 (3) p = 0.778	15.173 (6) **p = 0.019**
Glucose (log, AR-1)	305.61 (2) p < 0.001	57.532 (1) p < 0.001	3.187 (3) p = 0.364	56.184 (2) p < 0.001	4.174 (6) p = 0.653	1.642 (3) p = 0.650	4.632 (6) p = 0.592
Fructose (log, AR-1)	119.01 (2) p < 0.001	17.508 (1) p < 0.001	0.732 (3) p = 0.866	27.105 (2) p < 0.001	1.614 (6) p = 0.952	0.342 (3) p = 0.952	3.891 (6) p = 0.691
**Pinitol** (log, AR-1)	111.598 (2) p < 0.001	12.916 (1) p < 0.001	6.492 (3) p = 0.090	22.868 (2) p < 0.001	40.269 (6) **p < 0.001**	6.140 (3) p = 0.105	16.134 (6) **p = 0.013**
Raffinose (log)	30.447 (2) p < 0.001	67.415 (1) p < 0.001	0.648 (3) p = 0.885	30.156 (2) p < 0.001	3.723 (6) p = 0.714	4.952 (3) p = 0.175	3.852 (6) p = 0.697
Sucrose (log)	73.918 (2) p < 0.001	4.060 (1) p = 0.044	0.824 (3) p = 0.844	7.930 (2) p = 0.019	2.380 (6) p = 0.882	0.927 (3) p = 0.819	4.014 (6) p = 0.675
Xylose	31.003 (2) p < 0.001	1.075 (1) p = 0.300	3.064 (3) p = 0.382	56.060 (2) p < 0.001	2.248 (6) p = 0.896	2.020 (3) p = 0.568	5.724 (6) p = 0.455

Analysis of deviances for the hypothesis tested (organ, sampling date and snow manipulation treatment as the main effects and their two- and three-way interactions) for NSCs. The chi-squared values, the degrees of freedom (in the parenthesis) and the corresponding p-value are presented. If the response in the models is log-transformed, log is mentioned in the parenthesis Autoregressive (AR-1) structure is mentioned, if it has been used in the models (correlation structure between needles, stem, and roots of the same seedling). The NSCs with significant (< 5% risk level) treatment effect and/or its significant interactions are printed in bold.

### Chlorophyll fluorescence recovered from winter depression in spring

The photochemical efficiency of photosystem II, as measured by Fv/Fm ratios, highlighted the seasonal modulation of photosynthetic activity in Scots pine. On the first sampling date, the reduced Fv/Fm values (mean ± SE as 0.480 ± 0.017 over all treatments) in needles indicated down-regulation of light reactions in all the snow manipulation treatments (χ2 = 3.665, df=3, p=0.300). Fv/Fm was slightly lower in IE and NoSNOW than in AMB and COMP because of the decrease in both Fm and F0 values (data not shown) suggesting that there was somewhat more sustained quenching of fluorescence and stress under those treatments. All seedlings recovered from the Fv/Fm decline by the second sampling date (mean ± SE as 0.727 ± 0.009) indicating that sustained winter quenching had relaxed and photosynthesis reactivated (**Supplementary Figure 11**).

### IE, NoSNOW and COMP increased physical damage and mortality of Scots pine seedlings

On the first sampling date, apical buds were monitored for damages caused by different snow conditions (**Supplementary Figure 3**). All the apical buds were found to be either healthy or only slightly damaged in AMB. The most serious damages were caused by the IE treatment in which 60% of apical buds were heavily damaged, whereas in the NoSNOW and COMP treatments no more than 20% of apical buds were classified as heavily damaged (F=2.96, df=3/29, p=0.049; **Supplementary Figure 12A**). Seedlings were exposed to stronger stresses under the IE, NoSNOW and COMP treatments than in the AMB conditions also based on their health status during the following growing season. In AMB no dead seedlings were found, whereas under the snow manipulation treatments at least 20% of the seedlings were dead (F=6.44, df=3/154, p<0.001; **Supplementary Figure 12B**). However, seedling growth during the next growing season was not significantly affected by the snow manipulation treatments (χ2 = 1.22, df=3, p=0.75; **Supplementary Figure 12C**). Note however, that seedling growth was not measured in dead seedlings.

## Discussion

Snow conditions are changing due to climate warming. In this study, we found out that depending on the thickness and quality of the snow cover Scots pine seedlings were exposed to various abiotic stresses, whose effects were not limited to overwintering but extended also to the beginning of the following growing season. The snow manipulation treatments had impacts on several environmental factors such as ground level temperature, ground frost and subnivean gas concentrations, which led to altered gene expression in Scots pine seedlings. In the comparison between the snow manipulation treatments, it is important to bear in mind that seedlings were exposed to winter stresses also in the AMB conditions. This was indicated, for example, by remarkable *ADH* expression, which has been connected to anaerobic (Harry and Kimmerer, 1991) and low temperature (Jarillo et al., 1993) stresses. Thus, even small changes in stress levels between the treatments may be important for seedlings’ success. Generally, stress related genes showed strong expression on the first sampling date, but their expression decreased and the differences between the treatments levelled off by the second sampling date. Consistently, the great differences in carbohydrate metabolism and chlorophyll fluorescence between the sampling dates indicated an intense change in the physiological status of the seedlings during growth resumption.

In the NoSNOW treatment, strong *DHN3* and *LEA* expression indicated that without protective snowpack seedlings suffered from cold and/or drought stress. LEA proteins are well-known osmoprotectors (Battaglia et al., 2008), which accumulate under desiccation conditions in higher plants (van den Dries et al., 2011), including pines (Watkinson et al., 2003; Perdiguero et al., 2012). Dehydrins belong to group II LEA proteins, which are considered stress proteins involved in the formation of plants’ protective reactions to dehydration and cold (Close, 1997; Liu et al., 2017). Moreover, NoSNOW induced strong expression of *GOLS2*, which encodes a key enzyme in the synthesis of raffinose family oligosaccharides that function as osmoprotectants in plant cells (Nishizawa et al., 2008). However, expression in most of the genes was downregulated in NoSNOW compared to the other treatments. This was probably caused by freezing temperature which typically decreases the rate of enzymatic activity and thus disrupts metabolic processes and lead to general metabolic suppression (Beck et al., 2007). Decreased *CAT* expression agrees with the view that in Scots pine cells CAT protection against H_2_O_2_ damage is connected to active metabolism (Muilu-Mäkelä et al., 2015; Vuosku et al., 2015). Likewise, the expression of the glycolysis related *PK* gene decreased. In addition to cold, seedlings were also exposed to more intense sunlight in early spring. At the ground level the daily mean temperature was above zero for over two weeks before the ground surface started to thaw. Therefore, air temperature might reach the level when stomata opened in needles but there was no liquid water available as the ground was still frozen, which might lead to combined effects of stresses caused by the interaction of light, frost and desiccation in NoSNOW.

Although the IE treatment caused the most severe physical damage to the seedlings (see also Martz et al., 2016), gene expression in IE differed the least from the AMB conditions on the first sampling date. However, the difference in gene expression increased by the second sampling date, which suggested that IE caused delayed effects to the seedlings. Alcoholic fermentation was activated in needles on the first sampling date, which was indicated by increased expression of the genes coding the key enzymes pyruvate decarboxylase (PDC) and alcohol dehydrogenase (ADH) under the IE treatment. On the second sampling date, the expression of *βG* was downregulated in IE relative to AMB. Beta-glucosidases have various functions in plants, such as cell wall modification, defense, phytohormone signaling, secondary metabolism and lignification (Singh et al., 2016). Furthermore, the IE gene expression profile was generally characterized by drought stress related responses such as upregulation of the synthesis of soluble sugars (*SUS*, *GOLS2*), starch breakdown (*AMY1*) and production of osmoprotectants (*DHN3*, *LEA*) as well as downregulation of starch synthesis (*WAXY1*, *SS3*). Altogether, the results suggest that IE caused immediate hypoxic stress. Most likely IE also caused root damage which led to reduced water uptake and further a decrease in the water supply of needles in the beginning of the growing season but did not, however, decrease seedlings growth. Our findings are in line with the results of a previous dasotron experiment, which showed that in Scots pine seedlings the initiation of fine root growth was delayed by freezing of waterlogged soil, but root growth recovered later during the growing season (Roitto et al., 2019).

The COMP treatment simultaneously decreased snow depth and insulation capacity and restricted soil-atmosphere gas exchange. In the seedlings snow compaction increased the expression of the *MSHMT*, *CAT* and *ATG8* genes, which are connected to oxidative stress. MSHMT functions in the photorespiratory pathway which plays an important role in stress responses for preventing ROS accumulation in green tissues (Moreno et al., 2005; Voss et al., 2013). Catalase enzymes protect cells from the toxic effects by the conversion of H_2_O_2_ into water and molecular oxygen (Mhamdi et al., 2010). ATG8 is involved in the macroautophagy which is upregulated in response to stress conditions to degrade cytoplasmic components for providing raw materials and energy, and to eliminate damaged or toxic components that can be generated because of ROS accumulation (Pérez-Pérez et al., 2012; Avin-Wittenberg, 2019). In addition, the COMP treatment induced strong *GOLS2*, *PDC* and *ADH* expression. Altogether, our results suggest that the combination of cold and hypoxic stresses under the COMP treatment evoked different transcriptional responses than hypoxic and cold stresses under the IE and NoSNOW treatments, respectively. Previously, only a few studies (e.g. Saarinen et al., 2016) have included snow compaction in their experimental design. However, the strong and specific impact of snow compaction to both conifer seedlings and soil processes observed in our present and previous studies (Martz et al., 2016; Stark et al., 2020) urges for additional studies considering this change in the snow cover.

The snow manipulation treatments altered the expression of the phytochrome (*PHYO*, *PHYN*), cryptochrome (*CRY1*, *CRY2*) and clock component (*GI*, *TOC1*) genes on the first sampling date suggesting the cooperation of photoreceptors and the circadian clock system in adaptation to snow conditions. Especially, decreasing in expression of both photoreceptors and circadian clock genes in the NoSNOW treatment was obvious. In addition to reading the ratio of red to far-red light, PHYs function as thermosensors (Jung et al., 2016) and are involved in the regulation of cold tolerance (He et al., 2016) in angiosperm species. Also, the blue light-cryptochrome system has been connected to improved freezing tolerance by the effects on the plasma membrane stability in Arabidopsis (*Arabidopsis thaliana* L.). Imai et al. (2021) suggested that the plasma membrane in the *cry1cry2* mutants was more stable than in wild type plants because of the accumulation of dehydrins. The low expression of the photoreceptor genes as well as strong expression of the *DHN3* and *LEA* genes detected in Scots pine seedlings under the NoSNOW treatment agree with those previous findings in angiosperms. Instead, *GI* expression was induced by cold stress in wild type Arabidopsis plants, whereas *gi-3* mutants showed an increased sensitivity to freezing stress (Cao et al., 2005). However, the temperatures used for Arabidopsis were mildly cold compared to the extreme cold temperatures (even almost -30°C) experienced by Scots pine seedlings during the winter without a protective snowpack in the present study. Thus, the decrease detected in GI expression in the NoSNOW treatment was probably connected rather to dehydration than to cold acclimation. In addition to GI (Li et al., 2016), also phytochrome B (Liu et al., 2012) and TOC1 (Legnaioli et al., 2009) have been shown to be negative regulators in the response to dehydration conditions.

The circadian clock controls the annual growth cycle in trees growing in boreal regions by coordinating diverse physiological events with seasonal climatic changes in adaptive responses that are essential for survival (Singh et al., 2017). In Scots pine differences in the allele frequencies (Kujala and Savolainen, 2012) and expression levels (Alakärppä et al., 2018) of the circadian clock related genes between geographical areas indicate the importance of circadian clock in adaptation to local climate and photoperiod. Our present findings broadened the essential role of circadian clock in Scots pine adaptation to unpredictable stresses. In plants, a large proportion of clock-controlled genes are linked to stress regulation including genes with roles under cold, drought and osmotic stresses (Kreps et al., 2002; Sanchez et al., 2011), which is in line with our observations.

The snow manipulation treatments had only limited effects on the NSC metabolism. On the first sampling date, in NoSNOW the expression of the starch synthase gene (*SS3*) was downregulated as well as the expression of the *B3GALT* gene in the N-glycan processing pathway, which has been connected to cellulose biosynthesis (Nagashima et al., 2018). The result suggests that the production of soluble sugars was preferred because of their function as osmoprotectants and compatible solutes to mitigate the negative effects during cell dehydration (Thalmann and Santelia, 2017; Gangola and Ramadoss, 2018). However, no differences were found at the metabolite level in the NSC metabolism between the snow manipulation treatments unlike in a previous dasotron experiment, in which sucrose accumulated and the amount of starch reduced in the needles of Scots pine seedlings during the resumption phase after winters without snow or in flooded soil (Domisch et al., 2018). In pines frost hardening causes a strong suppression of the whole-plant net carbon uptake simultaneously with the induction of apical bud dormancy and growth cessation during cold acclimation (Krivosheeva et al., 1996; Savitch et al., 2002; Öquist and Huner, 2003). Unlike adult pine trees, young Scots pine seedlings had small pools for NSCs storage, and preceding photosynthate may have been used to withstand cold stress regardless of the snow manipulation treatments, thus leading to no apparent changes in NSCs between the treatments. In addition, pines may contain substantial quantities of lipids in woody tissues which can serve as carbon storage (Hoch et al., 2003). As lipids were not measured in this study, we cannot rule out the effect of the snow manipulation treatments on the total carbon pool.

Chlorophyll fluorescence has been generally regarded as a tool for interpreting stress and the extent of inactivation damage incurred by PSII (e.g. Maxwell and Johnson, 2000; Fracheboud and Leipner, 2003). In the present study, increased stress under the snow manipulation treatments led to light down-regulation of the *F*v/*F*m values and Rubisco (*RBCS*) expression compared to the AMB conditions on the first sampling date. Photosynthesis recovered almost equally in all snow conditions by the second sampling date. The seasonal modulation of photosynthetic activity is well documented in Scots pine in which CO_2_ assimilation is downregulated during winter and then gradually upregulated during spring in response to warming (e.g. Leverenz and Öquist, 1987; Karpinski et al., 1994; Vogg et al., 1998; Linkosalo et al., 2014; Yang et al., 2020). Our findings are in line with the view that in Scots pine the wintertime inhibition of photosynthesis is not the occurrence of damage but a dynamic protective mechanism which promotes success in severe conditions (Huner et al., 1992; Vogg et al., 1998; Repo et al., 2006). Also, the proper timing of the recovery of photosynthesis in the beginning of the growing season may be critical for the adaptation to snow conditions because of the need to maximize the length of the growing season and to minimize damage from exposure to cold temperatures and high irradiance at the same time. However, in the present study the detection of exact time of the recovery would have needed more frequent measurements of the photosynthetic parameters.

We can conclude that depending on the thickness and quality of the snow cover Scots pine seedlings were exposed to stresses in a complex way, when several different abiotic stresses affected in combination concurrently or nonsimultaneously. The stress combination in the COMP treatment resulted in a new pattern of gene expression which could not have been predicted by studying either hypoxic or cold stress in isolation. In plants transcriptional, metabolic and physiological responses to stress combinations may differ from those which are observed when the same stresses are applied separately (Mittal et al., 2012; Zandalinas et al., 2018). Therefore, it is especially important to study plant stress responses in authentic field conditions. Altogether, our findings work towards better understanding of snow impact on young conifer seedlings and forest regeneration in northern ecosystems under changing climate.

## Data availability statement

The raw data supporting the findings of this study is available from the corresponding author upon request.

## Author contributions

JV mainly designed the study, performed the gene expression analyses, and acted as a principal author of the manuscript. PR and FM planned the experimental design and established the experiment. FM recorded the field data, the seedling condition and growth, performed the gas, photosynthetic and sugar measurements. VH performed most of the statistical analyses. PR, FM and VH took part in writing the manuscript. All authors contributed to the article and approved the submitted version.
