# Supplementary material for: Changing winter climate and snow conditions induce various transcriptional stress responses in Scots pine seedlings
Source: Front Plant Sci. 2022 Dec 9;13:1050903. doi: 10.3389/fpls.2022.1050903 (PMC9780549; doi:10.3389/fpls.2022.1050903)
Supplement: Supplementary file 1 [file DataSheet_1.pdf]

Changing winter climate and snow conditions induce various transcriptional stress responses in Scots pine seedlings

Supplementary Material

Contents

Supplementary Figures 2
Supplementary Figure 1. Treatment plot design. 2
Supplementary Figure 2. Snow cover manipulation experiment. 3
Supplementary Figure 3. Classification of apical buds. 4
Supplementary Figure 4. Snow depth and ground surface temperatures in winter 2014-2015. 5
Supplementary Figure 5. Snow depth, snow water equivalent (SWE) and snow-covered area during spring 2015. 6
Supplementary Figure 6. Depth of soil frost and unfrozen soil surface in winter 2014-2015. 7
Supplementary Figure 7. Soil oxygen (O2) and carbon dioxide (CO2) concentrations in winter 2014-2015. 8
Supplementary Figure 8. Heatmap analyses of snow cover manipulation induced changes in gene expression. 9
Supplementary Figure 9. Canonical correlation analysis (CCA) ordination plot of the association between *DHN3*, *GOLS2* and *PDC* expression and environmental variables. 10
Supplementary Figure 10. Effects of snow manipulation treatments on the contents of non-structural carbohydrates in Scots pine seedlings. 11
Supplementary Figure 11. Effect of snow manipulation treatments on chlorophyll fluorescence in Scots pine seedlings. 12
Supplementary Figure 12. Effects of snow manipulation treatments on health and growth of Scots pine seedlings. 13
Supplementary Tables 14
Supplementary Table 1. qPCR primers for the expression analyses of Scots pine genes 14
References 16

Supplementary Figures

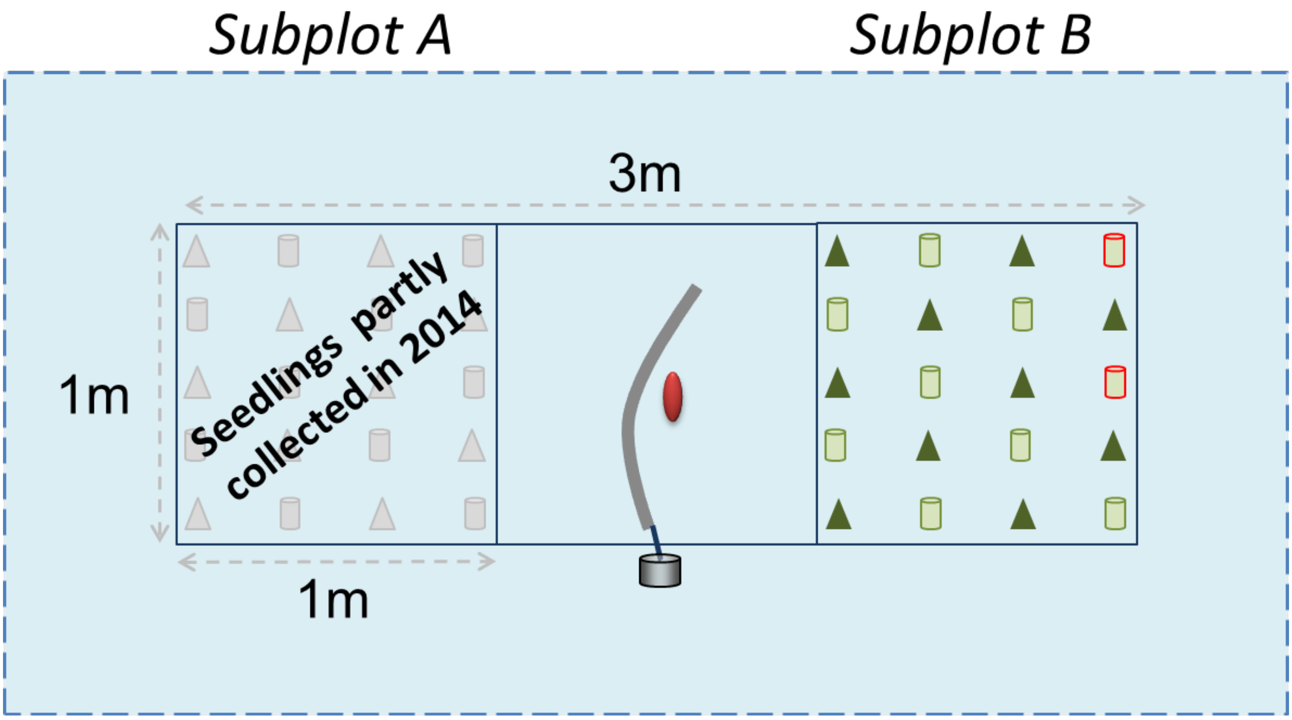

Supplementary Figure 1. Treatment plot design. Treatment plots (1 m x 3 m) were divided into three sections: two subplots (A and B, both 1 m x 1 m) separated by a buffer zone (1 m x 1m). The treated area (marked by blue colour) extended 0.5 m beyond the edges of the plot. Symbols: red dot = temperature logger; tube = air-collecting silicon tube inserted in humus layer; can = Scots pine seedling, can with red edges = Scots pine systematically collected for RNA and sugar analysis (unless damaged or dead); triangle = Norway spruce seedling. Seedlings were systematically collected from the right half of the subplot B, starting from the upper right corner. The remaining seedlings (maximum 8) were used for the growth and health inventory during the following growing season. Seedlings from the subplot A were previously used in Martz et al. (2016).

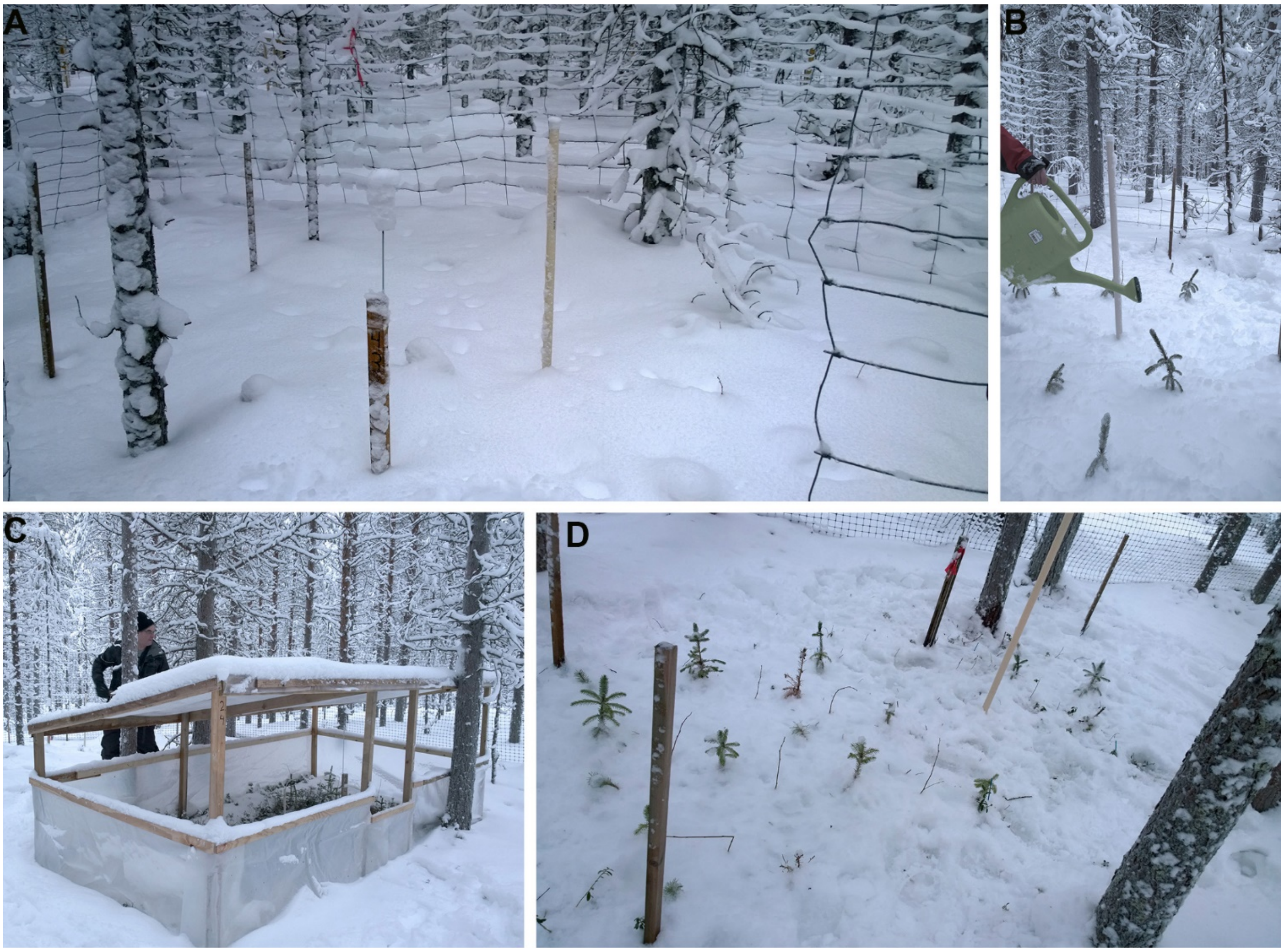

Supplementary Figure 2. Snow cover manipulation experiment. Ten randomized blocks, whose size varied from 100 to 200 m^2, were situated in the experimental field, which encompassed approximately three hectares as a whole. Blocks were situated so that the existing vegetation within each block was as homogenous as possible and fenced to exclude big herbivores. Four study plots were selected within each block and randomly assigned to the following treatments. (A) Ambient snow conditions (AMB) i.e. no treatment. (B) Ice encasement (IE) i.e. artificial formation of ice layers within the snow pack by two watering treatments on 8 January and 27 February 2015 using watering cans with thin roses. (C) No snow (NoSNOW) in which roofs and low walls made of translucent white plastic (thickness 0.2mm) were built over the plots to prevent snow fall and drifted snow. Shelters were set in place on 18 November 2014 and removed on 22 April 2015. Shelters had no effect on air temperature 30 cm above the soil surface in the middle of the plots (Martz et al., 2016 (D) Compaction of snow cover (COMP) in which snow was compacted by treading with hands at the same dates as snow was watered in IE.

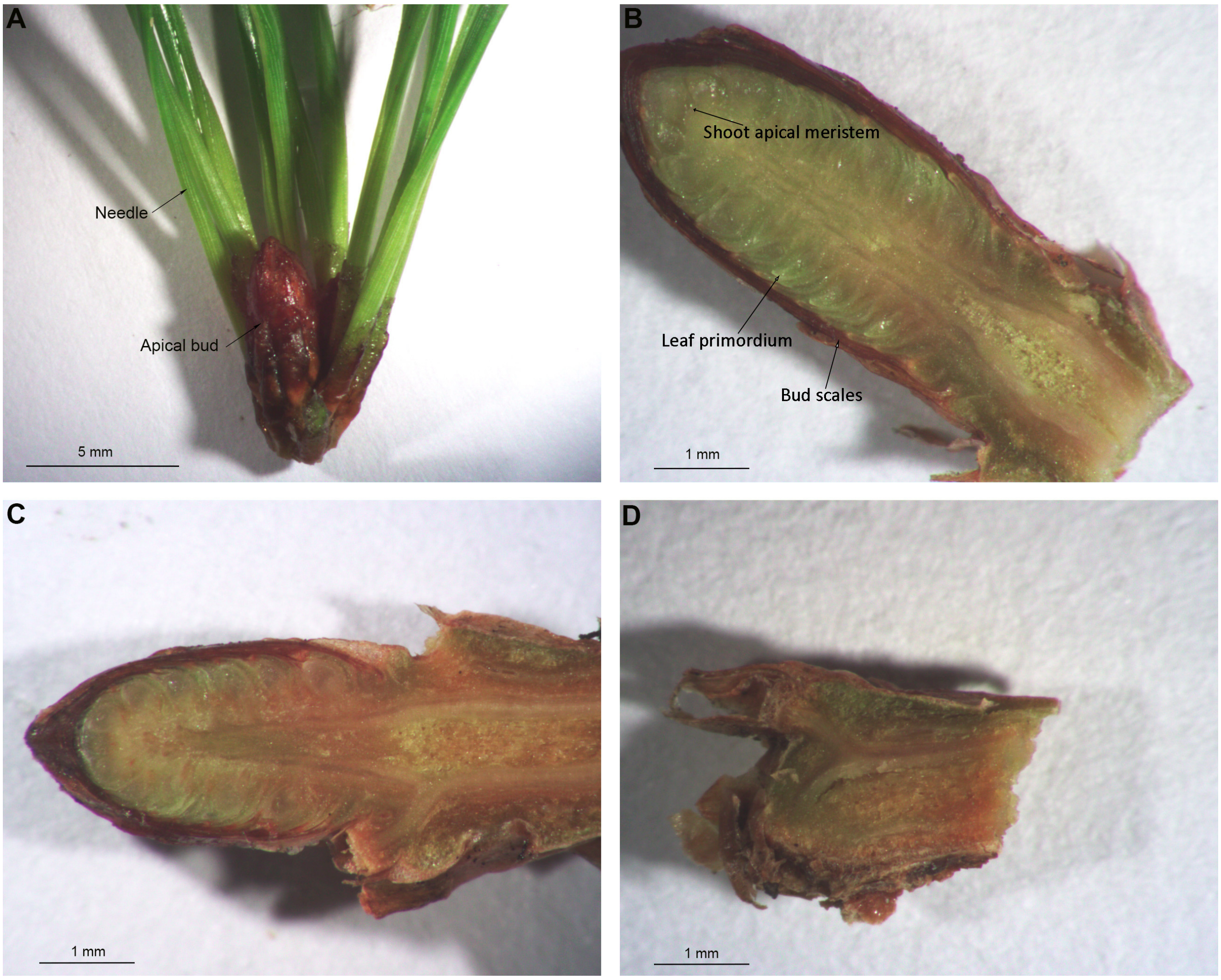

Supplementary Figure 3. Classification of apical buds. On the first sampling date (from May 4 to 7), the inner parts of the longitudinally cut apical buds were examined under a stereomicroscope to evaluate winter damage. Apical buds were classified as healthy, slightly damaged or heavily damaged according to their health. (A) Apical bud under stereomicroscope. (B) Longitudinally cut healthy apical bud with no visible damage. The shoot apical meristem is dormant during winter when it is protected by tightly closed bud scales and the developing leaf primordia. The shoot apical meristem becomes active in the beginning of the growing season, and after the bud scales drop off the leaf primordia develop into new needles. (C) Slightly damaged apical bud. (D) Heavily damaged apical bud.

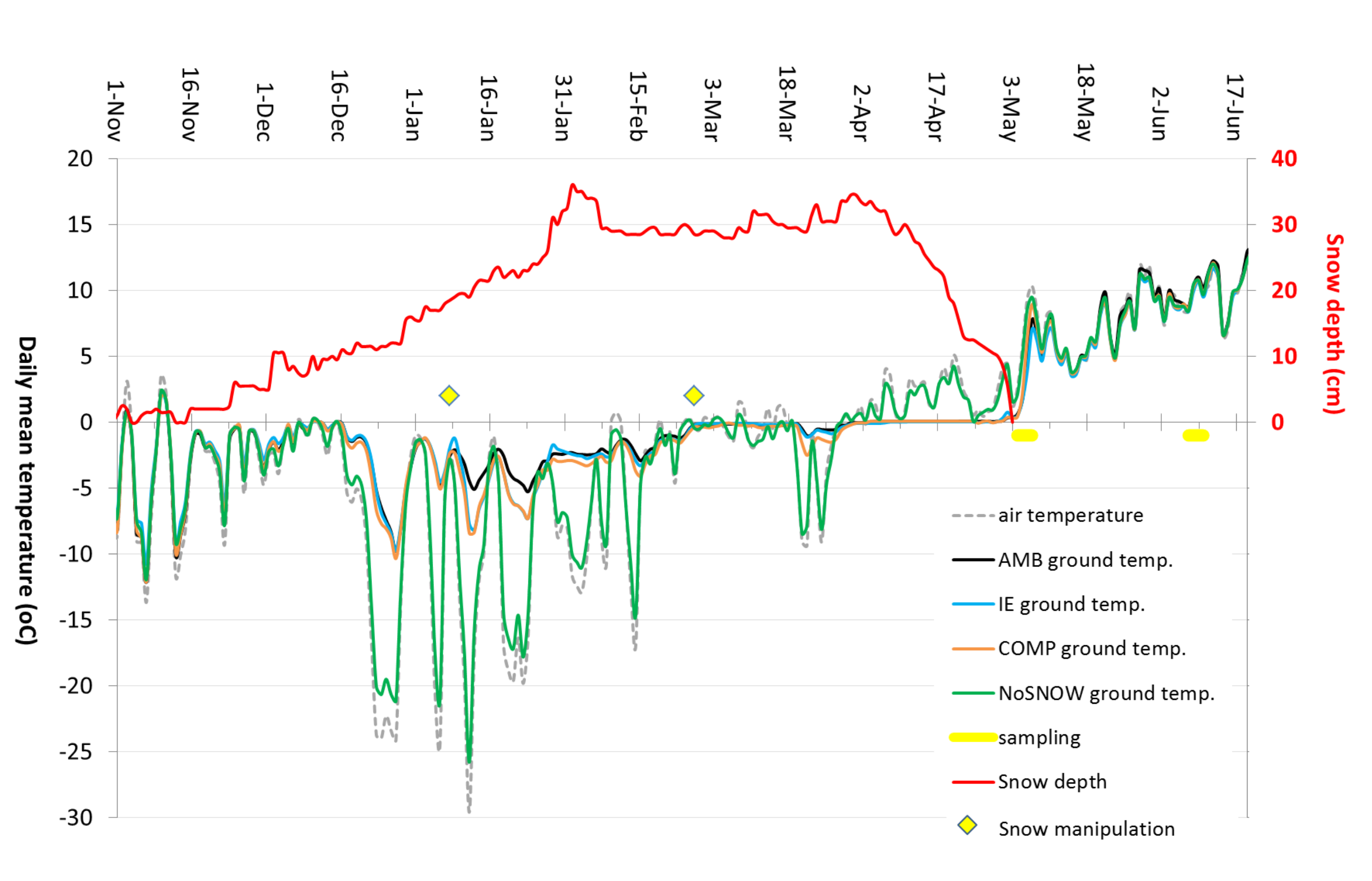

Supplementary Figure 4. Snow depth and ground surface temperatures in winter 2014-2015. Ground surface temperatures in ambient snow conditions (AMB) and under the ice encasement (IE), no snow (NoSNOW) and compacted snow (COMP) treatments are means of 4 measurements. Snow depth is based on the data provided by the Finnish Meteorological Institute (https://en.ilmatieteenlaitos.fi) from Apukka weather station (located 15 km north of Rovaniemi) and corrected to the forest experimental site (see also Martz et al., 2016, Stark et al., 2020).

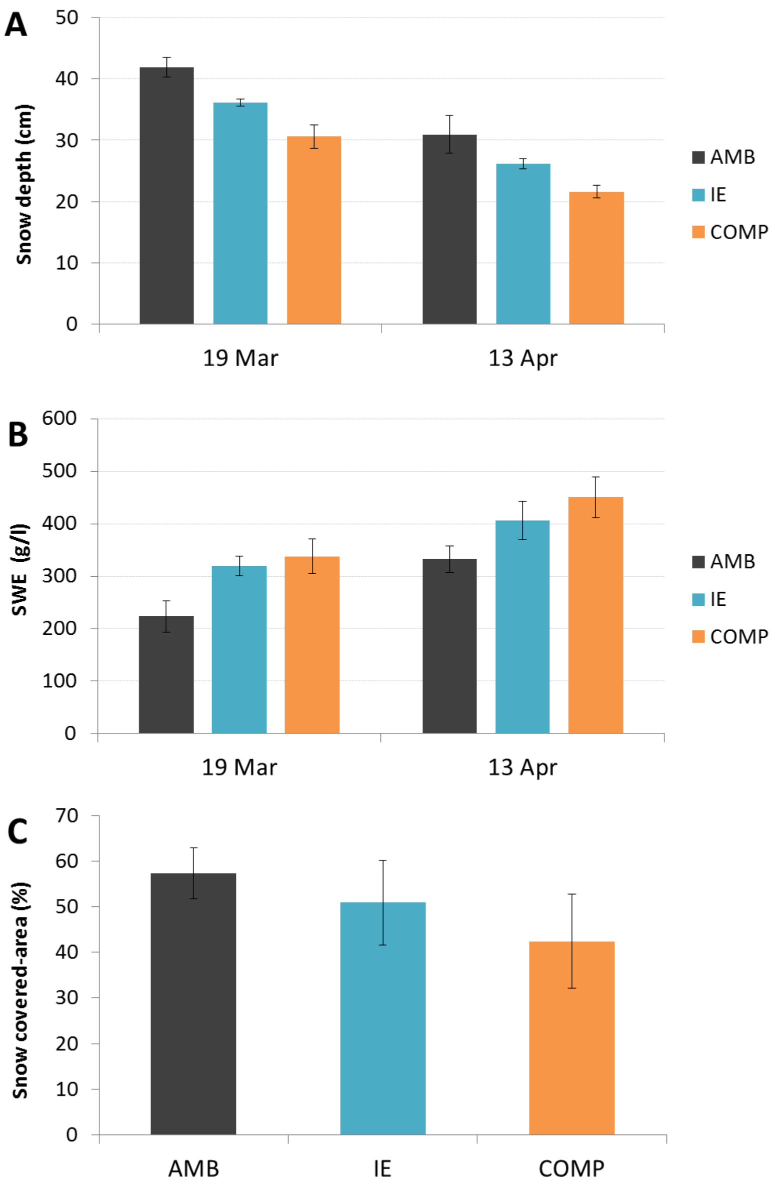

Supplementary Figure 5. Snow depth, snow water equivalent (SWE) and snow-covered area during spring 2015. (A) Snow depth (B) snow water equivalent and (C) snow-covered area in ambient snow conditions (AMB) and under the ice encasement (IE) and compacted snow (COMP) treatments on May 4, 2015. Values are mean ± SE (n=10).

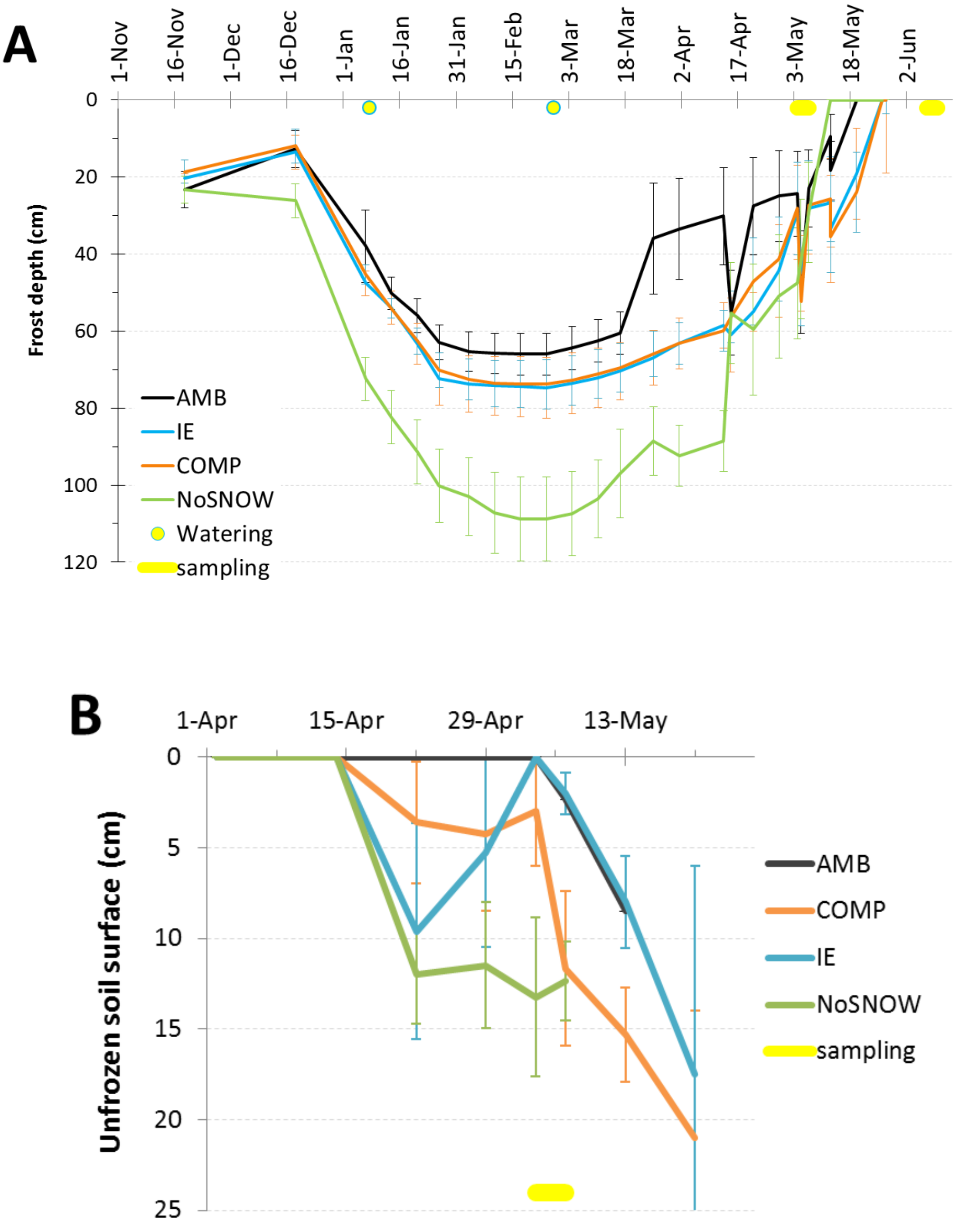

Supplementary Figure 6. Depth of soil frost and unfrozen soil surface in winter 2014-2015. During spring frozen soil thawed not only from the bottom but also from the surface. (A) soil frost depth and (B) the depth of unfrozen soil surface in the ambient (AMB) compacted snow (COMP), ice encasement (IE) and no snow (NoSNOW) treatment plots was measured using frost tubes filled with methylene blue dye (see Martz et al., 2016). Values are mean ± SE (n=5).

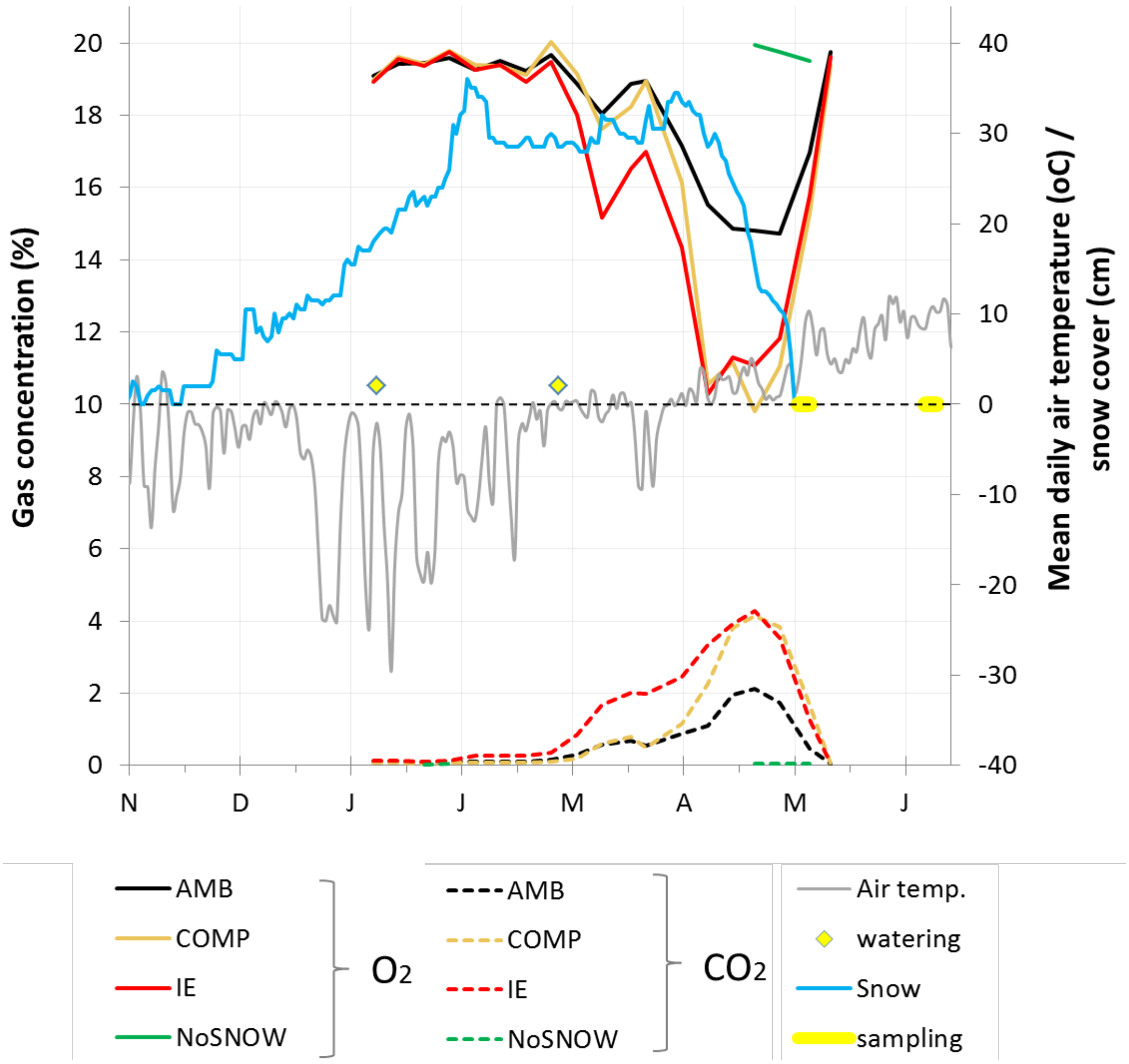

Supplementary Figure 7. Soil oxygen (O2) and carbon dioxide (CO2) concentrations in winter 2014-2015. Soil gas samples were collected from the ambient (AMB), compacted snow (COMP), ice encasement (IE) and no snow (NoSNOW) treatment plots using a syringe and silicone tubes buried into the humus layer and connected to the air via steep pipes (Martz et al., 2016). Samples were analyzed for O_2 and CO_2 by gas chromatography. Previous analyses indicated that soil gas concentrations in NoSNOW were similar as in the atmosphere and, therefore, gas samples were analyzed only occasionally in late spring (green lines). Values are means (n=10). For better clarity, the error bars are not included in the figure.

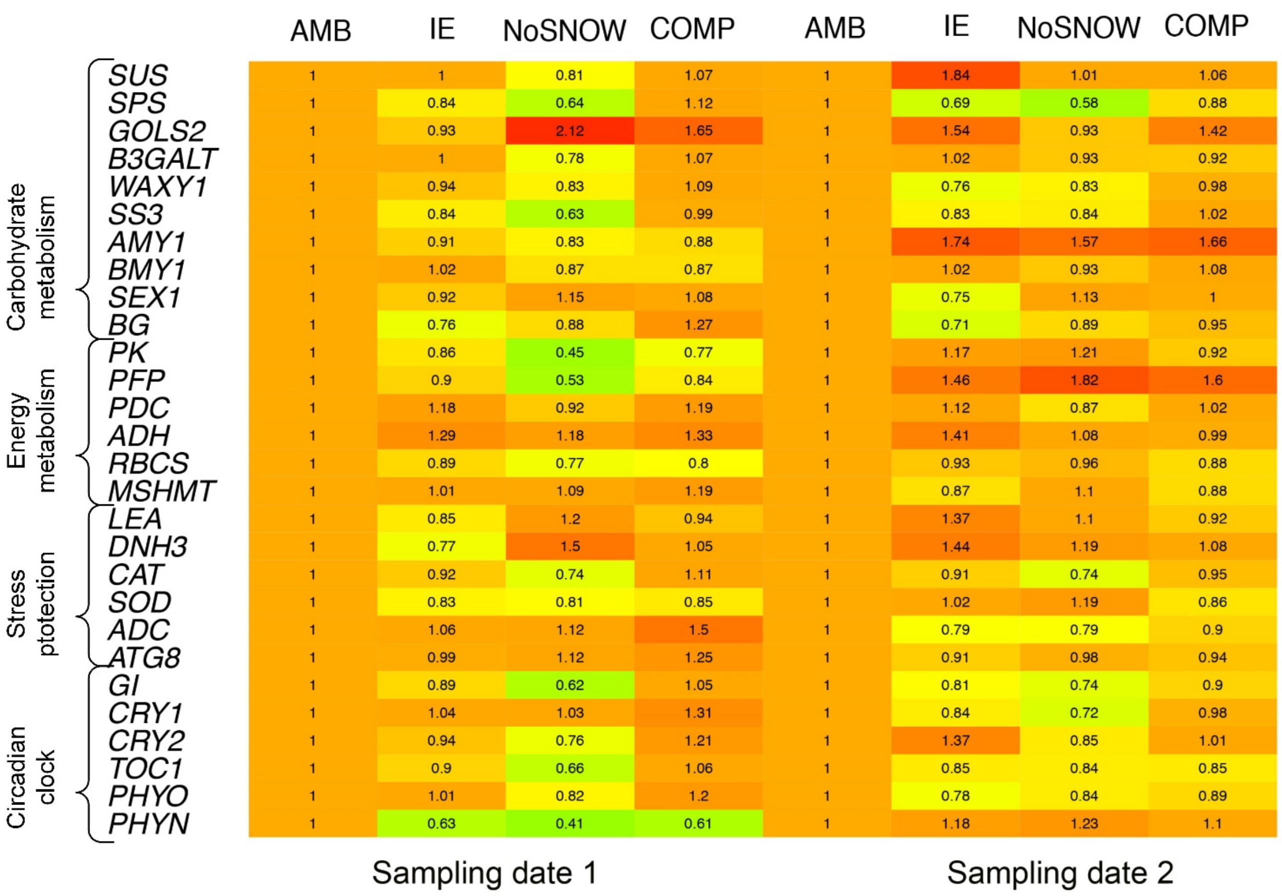

Supplementary Figure 8. Heatmap analyses of snow cover manipulation induced changes in gene expression. The genes were ordered into the functional groups: carbohydrate metabolism, energy metabolism, stress protection and circadian clock. The relative gene expression in the ice encasement (IE), no snow (NoSNOW) and compacted snow (COMP) treatments was compared to the expression in the ambient (AMB) separately on the first and second sampling dates. Thus, the values smaller and bigger than one indicate decreased and increased expression compared to AMB, respectively.

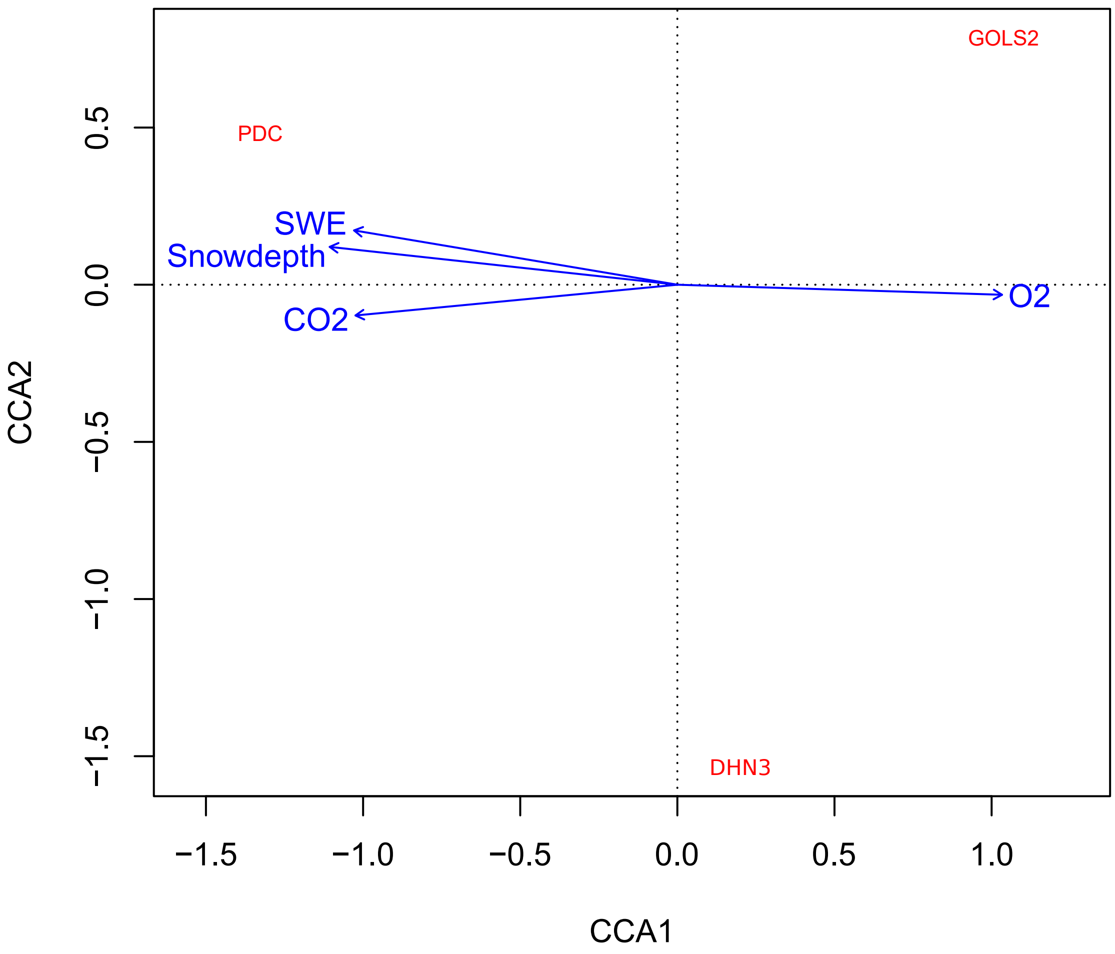

Supplementary Figure 9. Canonical correspondence analysis (CCA) ordination plot of the association between *DHN3*, *GOLS2* and *PDC* expression and environmental variables. The CCA ordination plot showing the first two principal dimensions of the relationship between the expression of the Scots pine dehydrin 3 (*DHN3*), galactinol synthase 2 (*GOLS2*) ja pyruvate decarboxylase (*PDC*) genes and the environmental variables on the first sampling date. The lengths and positions of the arrows provide information about the observed environmental variables and the derived axes. The mean concentration of oxygen in April (O_2) is related to the positive axis 1, and the mean snow water equivalent on April 13 (SWE), the mean snow depth on March 19 (Snowdepth) and the mean concentration of carbon dioxide in April (CO_2) are related to the negative axis 1. The projections of *PDC* onto the explanatory environmental variables suggests that *PDC* expression realizes high values of snow depth, SWE and CO_2 concentration, which are the cause of hypoxic stress. Instead, strong *DHN3* and *GOLS2* expression was more likely found in high O_2 concentrations in the absence of snow.

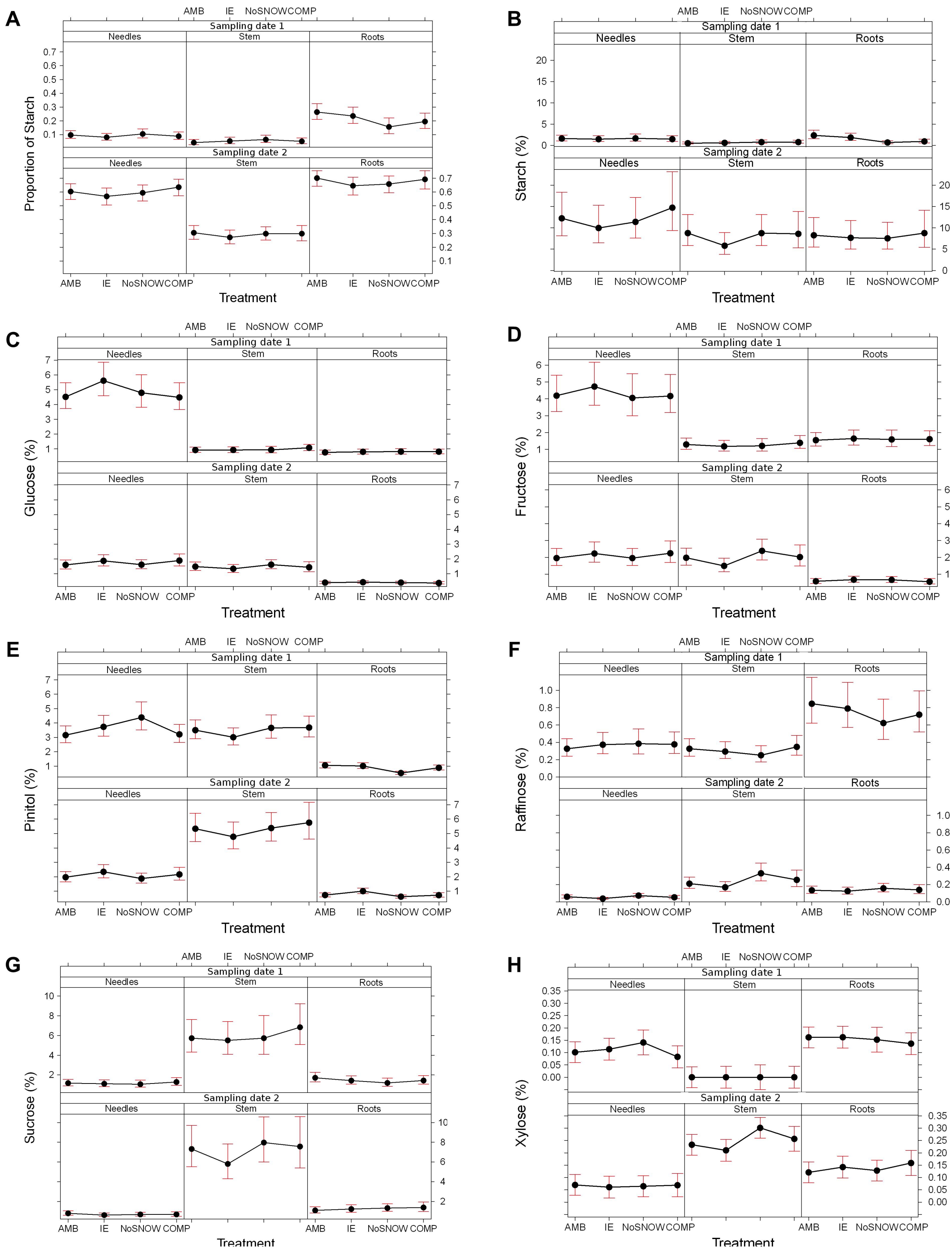

Supplementary Figure 10. Effects of snow manipulation treatments on the contents of non-structural carbohydrates (NSCs) in Scots pine seedlings. (A) The proportion of starch in the total amount of non-structural carbohydrates and (B) starch, (C) glucose, (D) fructose, (E) pinitol, (F) raffinose, (G) sucrose and (H) xylose concentrations were measured in the needles, stems and roots of the seedlings on the sampling dates 1 and 2. Seedlings had grown in ambient snow conditions (AMB) or under the ice encasement (IE), no snow (NoSNOW) or under compacted snow (COMP) treatment throughout two winters (n=10, 95% CIs).

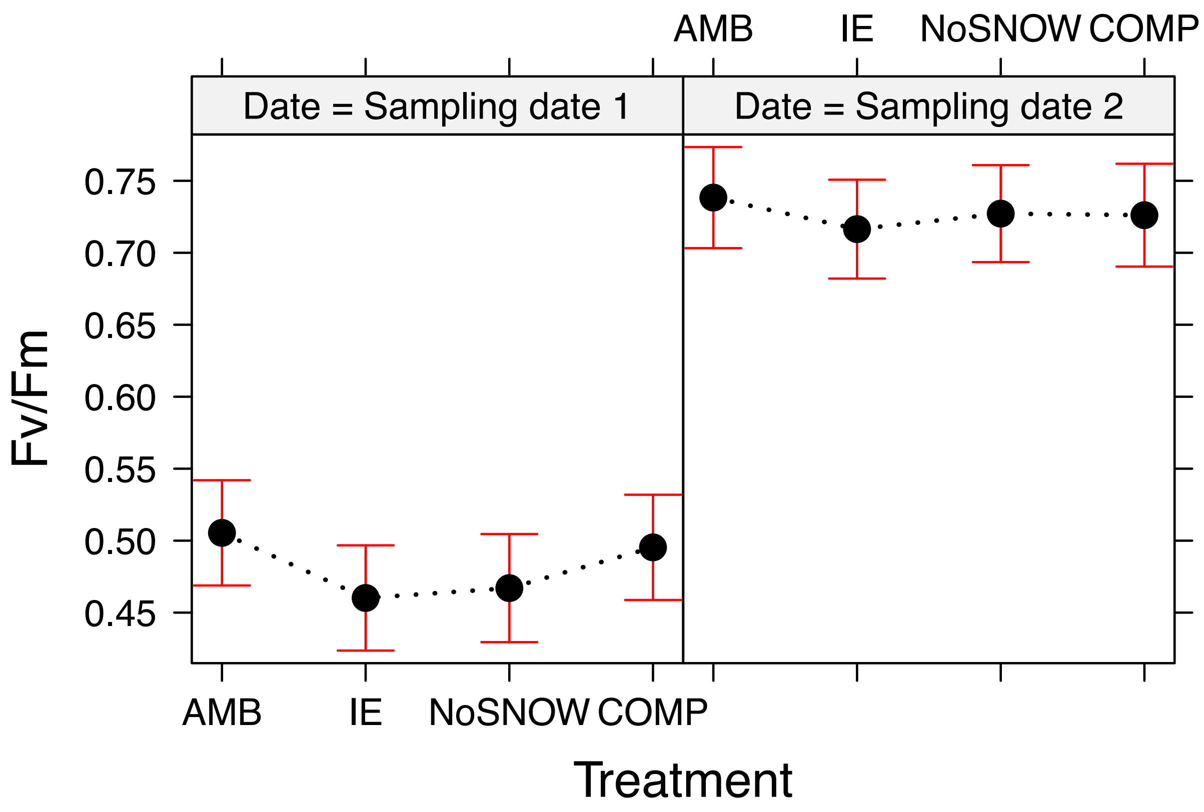

Supplementary Figure 11. Effect of snow manipulation treatments on chlorophyll fluorescence in Scots pine seedlings. The chlorophyll fluorescence parameter (Fv/Fm) reflecting the maximum quantum efficiency of photosystem II (PSII) photochemistry was measured from the current needles of Scots pine seedlings on sampling dates 1 and on 2. Seedlings had grown in ambient snow conditions (AMB) or under the ice encasement (IE), no snow (NoSNOW) or compacted snow (COMP) treatment throughout two winters (The total number of observations was 145 in the 10 blocks, 95% CIs).

A

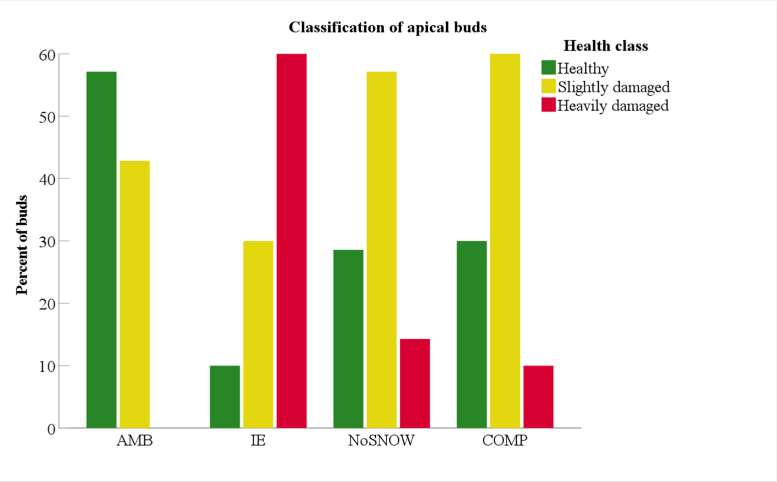

B

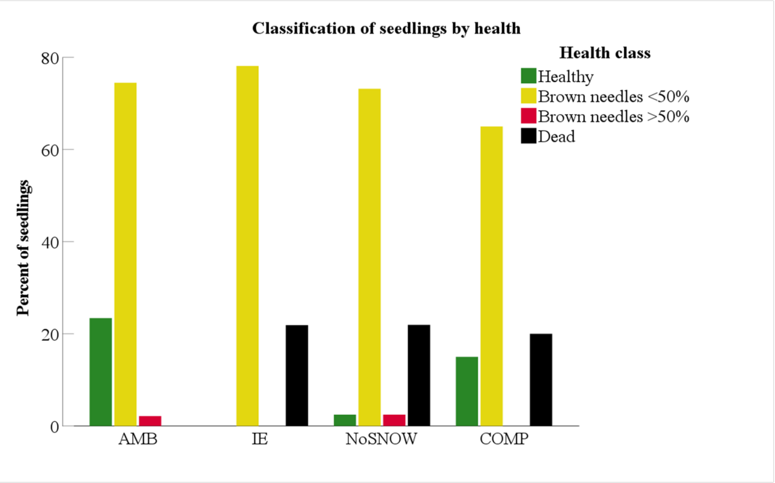

C

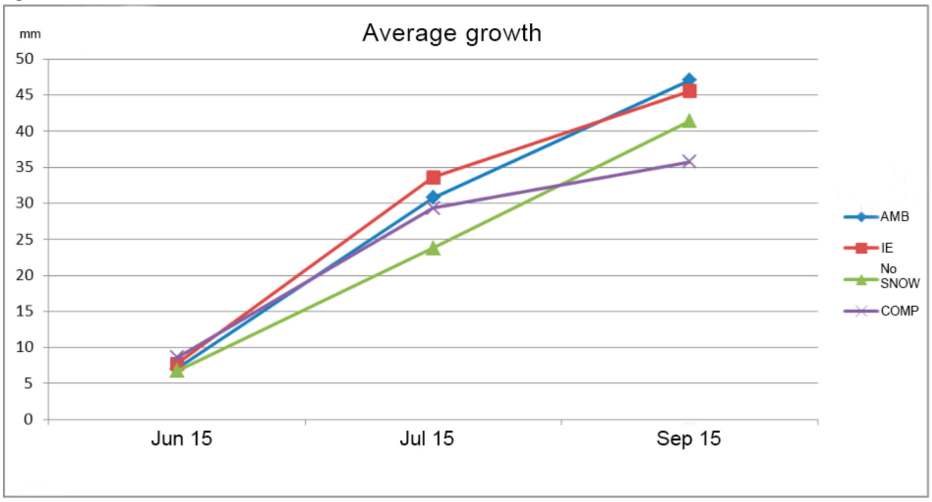

Supplementary Figure 12. Effects of snow manipulation treatments on health and growth of Scots pine seedlings. (A) The proportion of healthy, slightly damaged and heavily damaged apical buds on the first sampling date (n=10) (B) The proportion of healthy, damaged (classified by the amount of brown needles) and dead seedlings on July 15, 2015 (n=32-47 seedlings / treatment) (C) The average annual growth of the main shoot of the seedlings during the summer 2015 after the snow cover manipulation treatments (n=35-48 seedlings / treatment). Seedlings had grown in ambient snow conditions (AMB) or under the ice encasement (IE), no snow (NoSNOW) or under compacted snow (COMP) treatment throughout two winters.

Supplementary Tables

Supplementary Table 1. qPCR primers for the expression analyses of Scots pine genes

Gene	Sequence of the upstream PCR primer (5′-> 3′)	Sequence of the downstream PCR primer (5′-> 3′)	PCR product size (bp)	Genbank accession of the sequence
ACT	GGACAGGTCATTACCGTTGG	GATACCCGCTGCTTCCATT	90	M36171
ADC	AGTCCGTGTGGCCTGTAATC	TGCACAGACACAACGTCAAA	114	HM236823
ADH	AAGCAAAGGGTCAAGCTCCA	CCCGGCTTGAGGTGAGTTAC	100	FN824806
AMY1	GCCGAGGTATGACGTAGGAA	TTAGTCAGCGGAGGAGCTGT	109	PgdbPtadea_75082551*
ATG8	CCTGCTGATCTGACAGTTGGT	AGCAGCAGTTGGAGGTAGGT	114	KP864676
βG	CAAATCTGTTTGTGCCGTTG	CTGACGAGCAATTCCCTGTT	105	KM046994
B3GALT	ACTGCCAATCCTGCTACCAC	GTATGAACCTGCGGCGTACT	98	DR109699
BMY1	AAGCAAGTGATCCAGGCAAC	TTCTTCCAACCGAAGACTGG	123	GT258885
CAT	GGGAGGCAAACCTATGTGAA	TTGGTTGCATGACTGTGGTT	110	EU513163
CRY1	CTGCATTTTGGGGAGTTGAGTG	CCGGAGACCAATTGACTTCAGA	132	JQ969971
CRY2	TTCCCTGGCTGCAACAGAAA	CCCAACATTGCTAGGCAGGA	105	UCPtaeda_isotig18035
DHN3	CGGGACAACAGCAAAAGCTC	TTGTTTTGTTGTCCCGGCAG	282	AJ512362
GAPDH	CTGGTGTCTTCACCGACAAA	GGTGCTCATTAACCCCAACA	120	L07501

Gene	Sequence of the upstream PCR primer (5′-> 3′)	Sequence of the downstream PCR primer (5′-> 3′)	PCR product size (bp)	Genbank accession of the sequence
GI	ATTGCCATGGTCAGGTGGAG	AGGCCACATCTGATGCATCC	123	JQ969158
GOLS2	GGTGCAGAAATGGTGGGACA	TTTTCCGTCCGCCTCTGAAA	97	PgdbPtadea_4602
LEA	CTGGGTCAGTGAAGGCCAAT	TCCCAATCCCTTCCAACGTC	103	FJ201571
MSHMT	TGGTTCCAGGGTTGAAAGGG	ATGTCAAAGCTGGTGTCCCC	122	HE574554
PDC	TGTTGCTCCTACAGACCAGC	TGCTGAAACCGGTGACTCTT	112	CO161777
PFP	TGACTCTTGACACGCAACCT	CCACTTTTGTGCATTTTGGA	125	PgdbPtadea_44448
PHYN	GGAAGTTGAATTGGCTGCTCAG	TCTGAGTGACAATTCCCAAGGG	107	JQ970314
PHYO	GACGTCGAGGAAAATGTTGTGG	GGCCCTGTAATCACCTTGAAGA	105	EU120555
PK	TGTTGTGGTTCCTGTCCTGA	TTGCCTTTGCTGATCCTTCT	125	PgdbPtadea_34682553
RBCS	TGTGTGTATGTATGTGCGCG	ACCCAAACATCGGCAACTTC	111	AJ309096
SEX1	AGAGGAGAAGCGCTTGATTG	CCTGGGCACGTTTATAGAGC	128	PgdbPsylvestris_49374
SOD	GACATGCTGGGGATCTAGGC	CGAATGTGGCCCAGAGAGAG	98	X58578
SPS	GATCAAGCGGGTAAAGGTGA	ACCATCGGAACATTCAAAGC	110	AJ309090.1
SS3	CATTACTTGCTTGCACAACGA	CAGCCAAATGGGTTCAGTTT	123	GW762084.1
SUS	CCTGGTCTCTACCGTGTGGT	GTAAGGCGATGCTGCTTTTC	119	EF619967.1
TOC1	ACTCCAATACCAACAGTACCAA	ATATGTGAGGAAAGCTGATGC	210	JQ969596

Gene	Sequence of the upstream PCR primer (5′-> 3′)	Sequence of the downstream PCR primer (5′-> 3′)	PCR product size (bp)	Genbank accession of the sequence
UBI	GAAGGAGCAGTGGAGTCCTG	CAATTTCAGGGACGAGAGGA	104	AF461687
WAXY1	TGCGGTCTCATCCAGTTACA	ACCCATCTGGAACCCTGTTA	111	PgdbPsylvestris_53849

References

Martz, F., Vuosku, J., Ovaskainen, A., Stark, S., Rautio, P. (2016) The snow must go on: ground ice encasement, snow compaction and absence of snow variably cause soil hypoxia, CO_2 accumulation and tree seedling damage in boreal forest. *PLoS ONE*, 11:6.

Stark, S., Martz, F., Ovaskainen, A., Vuosku, J., Männistö, M.K., Rautio, P. (2020) Ice-on-snow and compacted and absent snowpack exert contrasting effects on soil carbon cycling in a northern boreal forest. *Soil Biol Biochem* 150, 107983.
